# Noncoding mutations cause super-enhancer retargeting resulting in protein synthesis dysregulation during B cell lymphoma progression

**DOI:** 10.1038/s41588-023-01561-1

**Published:** 2023-12-04

**Authors:** Rebecca J. Leeman-Neill, Dong Song, Jonathan Bizarro, Ludivine Wacheul, Gerson Rothschild, Sameer Singh, Yang Yang, Aditya Y. Sarode, Kishore Gollapalli, Lijing Wu, Wanwei Zhang, Yiyun Chen, Max C. Lauring, D. Eric Whisenant, Shweta Bhavsar, Junghyun Lim, Steven H. Swerdlow, Govind Bhagat, Qian Zhao, Luke E. Berchowitz, Denis L. J. Lafontaine, Jiguang Wang, Uttiya Basu

**Affiliations:** 1 Department of Microbiology and Immunology, Vagelos College of Physicians and Surgeons, Columbia University, New York City, NY USA; 2 Department of Pathology and Cell Biology, Vagelos College of Physicians and Surgeons, Columbia University, New York City, NY USA; 3 SIAT-HKUST Joint Laboratory of Cell Evolution and Digital Health, Shenzhen-Hong Kong Collaborative Innovation Research Institute, Shenzhen, China; 4 Division of Life Science, Department of Chemical and Biological Engineering, and State Key Laboratory of Molecular Neuroscience, The Hong Kong University of Science and Technology, Hong Kong SAR, China; 5 RNA Molecular Biology, Fonds de la Recherche Scientifique (F.R.S./FNRS), Université libre de Bruxelles (ULB), Biopark Campus, Gosselies, Belgium; 6 Institut für Medizinische Physik und Biophysik, Charité-Universitätsmedizin Berlin, Berlin, Germany; 7 State Key Laboratory of Chemical Biology and Drug Discovery, Department of Applied Biology and Chemical Technology, The Hong Kong Polytechnic University, Hong Kong SAR, China; 8 Department of Pathology, University of Pittsburgh School of Medicine, Pittsburgh, PA USA; 9 Department of Pharmacy, School of Pharmacy and Institute of New Drug Development, Jeonbuk National University, Jeonju, Republic of Korea; 10 Department of Genetics and Development, Vagelos College of Physicians and Surgeons, Columbia University, New York City, NY USA; 11 Hong Kong Center for Neurodegenerative Diseases, InnoHK, Hong Kong SAR, China

**Keywords:** B-cell lymphoma, Genomics

## Abstract

Whole-genome sequencing of longitudinal tumor pairs representing transformation of follicular lymphoma to high-grade B cell lymphoma with *MYC* and *BCL2* rearrangements (double-hit lymphoma) identified coding and noncoding genomic alterations acquired during lymphoma progression. Many of these transformation-associated alterations recurrently and focally occur at topologically associating domain resident regulatory DNA elements, including H3K4me3 promoter marks located within H3K27ac super-enhancer clusters in B cell non-Hodgkin lymphoma. One region found to undergo recurrent alteration upon transformation overlaps a super-enhancer affecting the expression of the *PAX5*/*ZCCHC7* gene pair. *ZCCHC7* encodes a subunit of the Trf4/5-Air1/2-Mtr4 polyadenylation-like complex and demonstrated copy number gain, chromosomal translocation and enhancer retargeting-mediated transcriptional upregulation upon lymphoma transformation. Consequently, lymphoma cells demonstrate nucleolar dysregulation via altered noncoding 5.8S ribosomal RNA processing. We find that a noncoding mutation acquired during lymphoma progression affects noncoding rRNA processing, thereby rewiring protein synthesis leading to oncogenic changes in the lymphoma proteome.

## Abstract (web summary)

Whole-genome analysis of paired follicular lymphoma and double-hit lymphoma shows that lymphoma progression is accompanied by enhanced somatic mutations targeting super-enhancer-embedded promoters.

## Main

B cells undergo a series of programmed genomic alterations that enable the immunoglobulin light and heavy chain loci to generate high-affinity antibodies against invading pathogens. First, B cells undergo variability, diversity and joining (VDJ) recombination in the bone marrow with subsequent somatic hypermutation (SHM) and class switch recombination (CSR) occurring within lymphoid follicles once the cells traffic to secondary or tertiary lymphoid organs^1,2^. Both CSR and SHM require the essential activity of the enzyme activation-induced cytidine deaminase (AID) that incorporates mutations via single-strand DNA nicks at variable region genes and introduces DNA double-strand breaks at switch sequences to initiate the process of SHM and CSR, respectively^3–6^. VDJ recombination, SHM and CSR all can also lead to DNA alterations outside the boundaries of the immunoglobulin gene loci, many of which promote lymphomagenesis^7,8^. The mechanism by which AID recognizes its target DNA sequences in the B cell genome is incompletely understood^5,9–14^. In this context, a better understanding of DNA targeting by AID, specifically in models of lymphoma progression, would be a significant advance. Furthermore, the consequences of AID-mediated nonimmunoglobulin locus-associated somatic mutation, so-called aberrant somatic hypermutation (aSHM) identified in mice and humans at coding and noncoding sequences, are only beginning to be evaluated^4,7,15^. The landscape of coding-region mutations observed in lymphoma does not account for the numerous alterations in gene expression required for lymphomagenesis. Therefore, it is possible that aSHM affecting gene regulatory regions greatly contributes to perturbations in gene expression at the transcriptional and translational levels, beyond altering single specific genes at or adjacent to the sites of aSHM. This role for aSHM could have important implications for our understanding of the pathophysiology of lymphoid malignancies and potentially neoplasia in general.

### Genomic alterations acquired during lymphoma transformation

**Extended Data Fig. 1:**
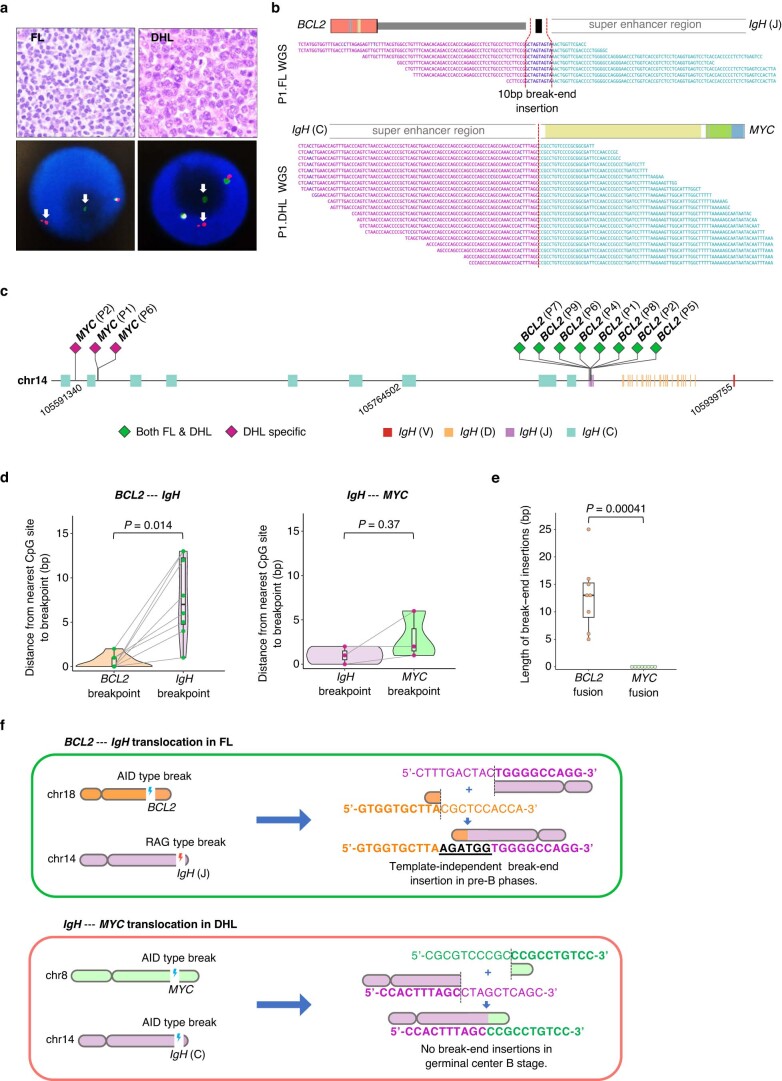
Features of *BCL2* and *MYC* alterations. **(a)** Examples of longitudinal samples of follicular lymphoma (FL, left) and double hit lymphoma (DHL, right) obtained from the same patient (100X) (top). Fluorescence in situ hybridization (FISH) performed on a DHL, using break-apart probes, showing rearrangements involving *BCL2* (left) and *MYC* (right) (bottom). **(b)** Examples of *BCL2* and *MYC* translocations identified via whole genome sequencing (WGS). The split reads that support *BCL2-IGH* in P1 show a 10bp break-end insertion. Supporting reads of *IGH-MYC* in P1 are fully mapped to the *IGH-MYC* junction. **(c)** *BCL2* and *MYC* translocation breakpoints in *IGH* loci. All three *IGH-MYC* breakpoints are located at the intergenic region of *IGH* C genes. All *BCL2-IGH* breakpoints are clustered within *IGH* J genes. **(d)** Sequence pattern analysis of *BCL2-IGH* (n = 8) and *IGH-MYC* (n = 3) breakpoints. *BCL2-IGH* breakpoints always locate no more than 2bp from CpG sites in *BCL2* but far from CpG sites in *IGH* loci, while breakpoints of *IGH-MYC* are close to CpG sites in both *IGH* and *MYC*. *P*-values were calculated by two-sided Wilcoxon signed rank tests. The boxplots display the 25^th^ and 75^th^ percentiles and median of each group. Whiskers represent highest and lowest values within 1.5 ${\rm{\times }}$ the inter-quartile range. **(e)** Comparison of break-end insertions between *BCL2* fusions (n = 8) and *MYC* fusions (n = 8). All *BCL2* translocations present template-independent break-end insertions with lengths ranging from 5–25 bp. None of the *MYC* translocations show break-end insertions. *P*-values were calculated by two-sided Wilcoxon rank-sum tests. The boxplots display the 25^th^ and 75^th^ percentiles and median of each group. Whiskers represent the highest and lowest values within 1.5 ${\rm{\times }}$ the inter-quartile range. **(f)** Models of *BCL2-IGH* in FL and *IGH-MYC* in DHL. Double strand breaks (DSB) in *BCL2* are caused by activation induced cytidine deaminase (AID) activity at CpG sites in follicular lymphoma and/or immature B cells and are joined with the breakpoints of *IGH* resulting from RAG activity during VDJ recombination. Subsequently, the break-end joining process is triggered by TdT, a specialized DNA polymerase that adds nucleotides at junctions. In DHL, AID-associated DSBs are detected in both *MYC* and *IGH* C genes. No break-end insertions are observed.

aSHM within both coding and noncoding regions has been observed in several classes of B cell non-Hodgkin lymphoma (B-NHL), particularly those originating from germinal center B cells^16,17^. Most low-grade B-NHLs are relatively indolent and, while often incurable, are not associated with heightened mortality. However, low-grade lymphomas can transform into more aggressive lymphomas^18^. For example, 25–35% of patients with low-grade follicular lymphoma (FL) experience transformation from a clinically indolent state to an aggressive and frequently fatal diffuse large B cell lymphoma (DLBCL)^19,20^. Prior genomic studies have investigated changes occurring upon FL transformation^21–27^. Several clinical and molecular prognostic indices for risk stratification of FL and prediction of transformation also have been proposed^28,29^. In our study, we have focused on transformation-associated DNA alterations observed in an important subset of B-NHL—‘double-hit’ lymphomas that harbor *MYC* rearrangements in addition to the *BCL2* rearrangement characteristically observed in FL and which are highly aggressive and difficult to treat (Extended Data Fig. 1a).

**Fig. 1:**
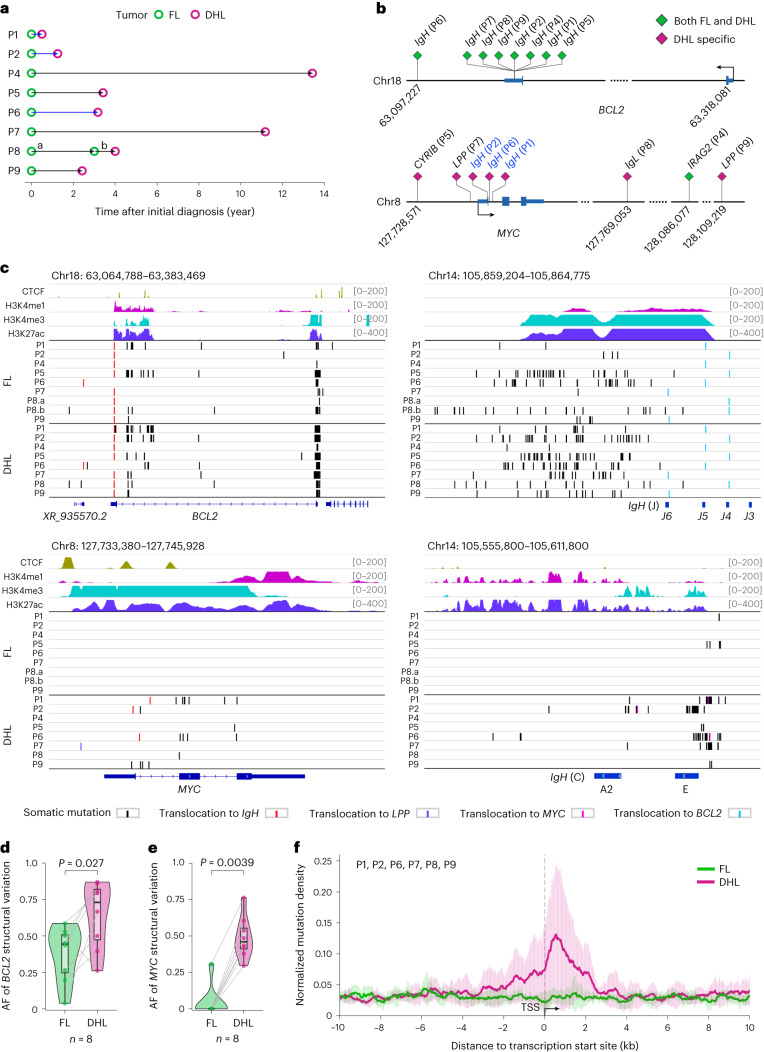
Whole-genome sequencing of longitudinal FL/DHL samples reveals transformation-specific genomic alterations. **a**, Eight cases of DHL with an available preceding FL sample diagnosed in the previous 17 years were identified. All DHL fulfilled WHO diagnostic criteria^66^, showing large B cell lymphoma morphology and harboring both *MYC* and *BCL2* translocations identified in fluorescence in situ hybridization studies. For all patients, the FL sample preceded the DHL sample by months to years (average time to transformation 59 months, range 6–161 months). For one patient (P8), two FL samples were available in addition to the subsequent DHL. **b**, Schematic describing the *BCL2* and *MYC* rearrangements observed for each patient. From WGS data, we identified all rearrangement breakpoints in the *BCL2* and *MYC* loci. On the *BCL2* locus, all tumor samples were found to harbor translocations to *IGH*, with breakpoints clustered in the 3′ UTR and downstream of *BCL2*. *MYC* rearrangements were mainly observed as DHL tumor-specific, with the exception of P4, which harbored an *MYC* translocation in both the FL and DHL samples. All three patients harboring *MYC-IGH* translocations (blue font) show breakpoints close to the first intron of the *MYC* gene. **c**, Mapping of *BCL2-IGH* and *MYC-IGH* translocations, and aSHM at *BCL2*, *MYC* and corresponding *IGH* loci. The occurrence of aSHM on *BCL2* and *MYC* was significantly associated with *IGH* as the translocation partner (two-sided Fisher’s exact test *P* = 1.27 × 10^−6^). **d**,**e**, Plots showing increasing AF for *BCL2* (**d**) and *MYC* (**e**) structural variants with transformation from FL to DHL (*n* = 8 patients). The *MYC* translocation in P4 was detected in both FL and DHL. *P* values were calculated by one-sided Wilcoxon signed-rank tests. The boxplots display 25th and 75th percentiles and the median of each group. Whiskers represent the highest and lowest values within 1.5× interquartile range. **f**, Comparison of mutational burden of FL compared to DHL in relation to the TSS, normalized and averaged over the cohort. A substantial concentration of mutations was observed within a 1,000 bp proximity of the TSS particularly within DHL. Only samples for which a nontumor DNA sequence was available are included in this analysis. AF, allele frequency. Source data

In using longitudinal samples from the same patient, we sought to characterize aSHM events occurring at different stages during lymphoma progression and identify mutations that appear specific to FL transformation as opposed to those incurred during the development of de novo DLBCL. Because transformation to double-hit lymphoma (DHL), by definition, includes acquisition of an AID-dependent *MYC* translocation^25^, we felt that the role of AID in lymphoma transformation would be well illustrated through these samples. We identified a series of eight patients (clinical information described in Supplementary Fig. 1) diagnosed with DHL and for which preceding FL specimens were also available, with time to transformation ranging from 6 to 161 months (Fig. 1a). Longitudinal FL/DHL samples and, when available, nontumor DNA from nonneoplastic specimens for the same patient (for example, bone marrow and appendix) were subjected to whole-genome sequencing (WGS; see Supplementary Tables 1–3 for mutation information). As expected, characteristic translocations of the 3′ end of *BCL2* to the *IGH* locus were detected in all lymphomas, with *MYC* translocations to various partner loci seen upon transformation to DHL (Fig. 1b). In addition to the acquisition of *MYC* translocations, transformation-specific changes included both increasing aSHM at the *BCL2* promoter and increasing variant allele frequency of the *BCL2* translocation observed in FL (Fig. 1c–e). Detailed evaluation identified break-end insertions at *BCL2* translocation breakpoints (with signature insertions via TdT enzyme) and blunt end joining at the *MYC* locus (Extended Data Fig. 1b–e), indicating that *BCL2* translocations are recombination activating gene (RAG)-endonuclease complex-dependent whereas *MYC* translocations are AID-dependent. These findings support the acquisition of *IGH-BCL2* translocations in immature, RAG-expressing B cells (Extended Data Fig. 1f) followed by subsequent oncogenic mutations and ultimately *MYC* translocation upon the development of DHL^30,31^.

### DHL-specific mutations occur within SE-embedded promoters

**Fig. 2:**
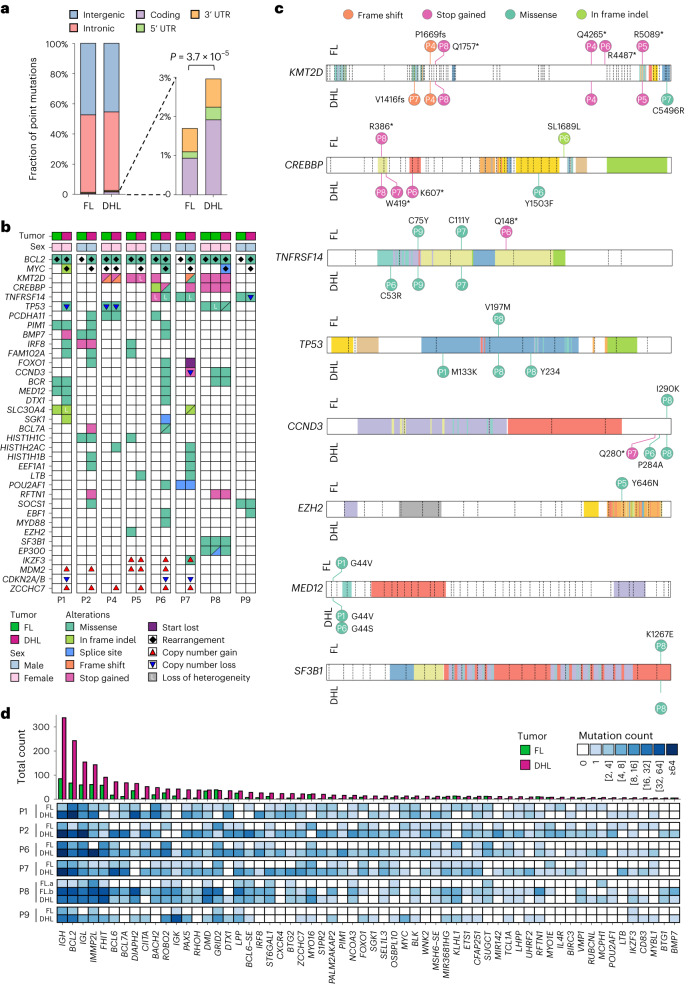
Coding and noncoding mutations occurring in FL and DHL. **a**, Percentages of somatic point mutations observed in coding and noncoding regions within FL and DHL (left) and breakdown of genic mutations observed (right), showing more frequent coding region and 5′ UTR mutations in DHL relative to FL. The *P* value was calculated by the two-sided chi-squared test. **b**, Summary of alterations in coding and noncoding regions surrounding highlighted genic sequences observed in the cohort, including single-nucleotide variants, short indels, copy number variants and structural variants. Genes included in this figure are selected based on mutation frequency, mutation impact, COSMIC annotation, OncoKB information and a published study of DLBCL genomes^16^. **c**, Lollipop plots of nonsynonymous mutations in well-recognized oncogenes and tumor suppressor genes. The asterisks indicate a translation stop codon. **d**, Noncoding mutations seen in the six patients that could be analyzed (due to availability of nontumor DNA sequences) showing consistently increasing mutational frequency in the noncoding (intronic or promoter associated) DNA sequences of each gene upon transformation. Source data

**Fig. 3:**
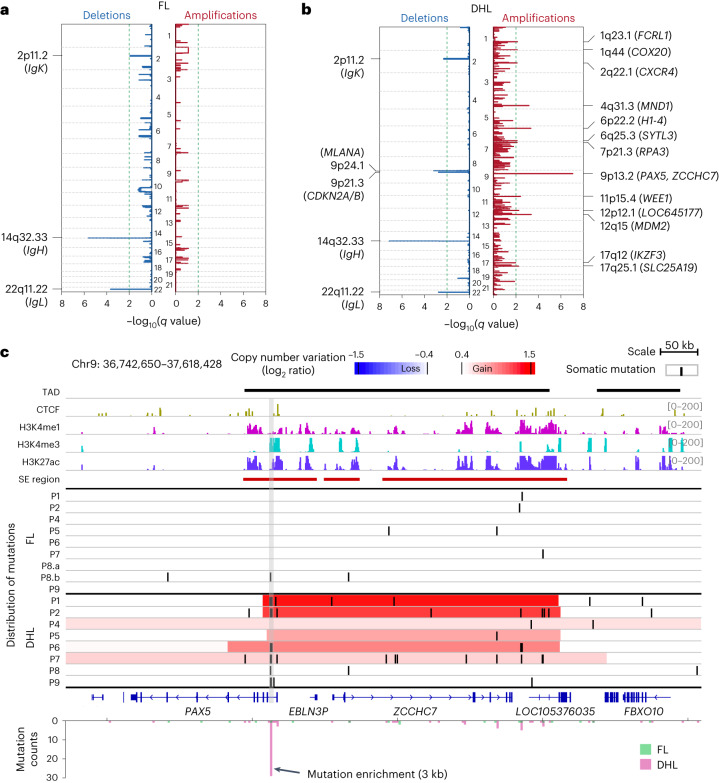
Transformation-specific copy number changes and other alterations at the PAX5/ZCCHC7 locus. **a**,**b**, Copy number changes, that is, losses and gains seen in the genomes of (**a**) FL and (**b**) DHL samples. The GISTIC plots show significant focal copy number variation (including recurrent *CDKN2A*/*B* loss, *MDM2* gain and *PAX5*/*ZCCHC7* gain) occurring during transformation to DHL. **c**, Copy number and single-nucleotide variants involving the *PAX5*/*ZCCHC7* locus. Most cases demonstrate a gain of *ZCCHC7* upon transformation to DHL (Fisher’s test *P* = 0.0023). TAD, CTCF, H3K4me1, H3K4me3 and H3K27ac ChIP–seq data overlap the *PAX5*/*ZCCHC7* locus. The area of copy number gain of *PAX5*/*ZCCHC7* mostly overlaps the entirety of the TAD. Transformation-associated aSHM at *PAX5* is enriched in the first intron and overlaps with H3K4me3 and H3K27ac peaks (gray vertical box). Source data

In mouse B cells, AID-associated chromosomal translocations occur at promoters and inside gene bodies^15^. By analyzing paired patient samples by WGS, we find that FL and DHL harbor mutations in both coding and noncoding sequences (Figs. 1f and 2a), with a higher coding-region mutational burden observed in DHL relative to FL (analyses in Fig. 2b). Many mutations are observed in known B-NHL oncogenes including *KMT2D*, *CREBBP*, *TNFRSF14*, *TP53*, *CCND3*, *EZH2*, *MED12* and *SF3B1* (Fig. 2c)^16,24,32–34^. In addition to coding-region mutations, numerous mutations are observed at noncoding DNA sequences (often intragenic but some intergenic). Strikingly, many mutations acquired upon transformation to DHL cluster specifically within 2 kb of the transcription start sites (TSS) of genes (Fig. 1f), including several previously found to be mutated in B cell lymphomas (Fig. 2d). Many of these mutations occur within noncoding sequences, often in the first intron of genes known to undergo AID-mediated aSHM (for example, aSHM at *MYC* and *BCL2* loci; Fig. 1c). In addition to single-nucleotide variants, we observe recurrent copy number gains at the *ZCCHC7*/*PAX5* and *MDM2* loci and recurrent losses at *CDKN2A*/*B* in DHL (Fig. 3a,b). Copy number gains at the *ZCCHC7*/*PAX5* locus are surprisingly recurrent, acquired upon transformation to DHL in 6 of 8 patients (Figs. 2b and 3c).

**Fig. 4:**
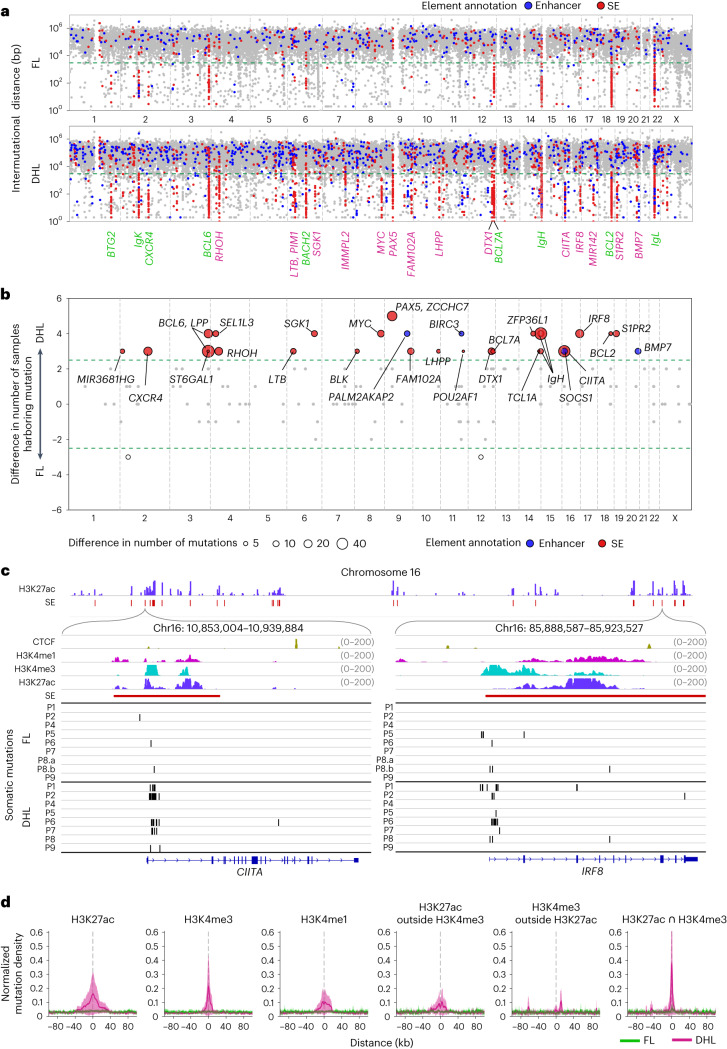
Enrichment of transformation-specific mutations in SE-embedded promoters. **a**, Rainfall plots of the intermutational distances across the genome in FL and DHL demonstrate that the majority of the mutation-enriched clusters, some showing a kataegis pattern, overlap SE regions. Hypermutated genes in both FL and DHL are labeled in green, and DHL-specific hypermutated genes are labeled in magenta. **b**, Unbiased analysis highlighting genes enriched for acquired mutations (mutations occurring upon transformation to DHL). After separating the genome into 3 kb segments and comparing the somatic mutation profile for DHL versus FL in high mutation load segments with at least five mutations, 30 of 204 segments show significantly more mutations in DHL. All these segments overlap with SE (labeled in red) or enhancer (labeled in blue) regions. These regions were then labeled with the names of nearby genes. **c**, Examples of regions recurrently mutated upon FL to DHL transformation. Both *CIITA* and *IRF8* show hypermutation clusters overlapping SE regions and close to the TSS. **d**, Normalized and averaged somatic mutation density of FL and DHL in relation to H3K27ac marks representing active enhancers, H3K4me3 marks representing active promoters near TSS and H3K4me1 marks representing primed enhancers. The three panels on the right show mutation density in H3K27ac regions outside H3K4me3 areas, H3K4me3 regions outside H3K27ac marks representing promoters outside enhancers and intersection areas of H3K27ac marks and H3K4me3 marks representing promoters present within active enhancer regions. Source data

**Extended Data Fig. 2:**
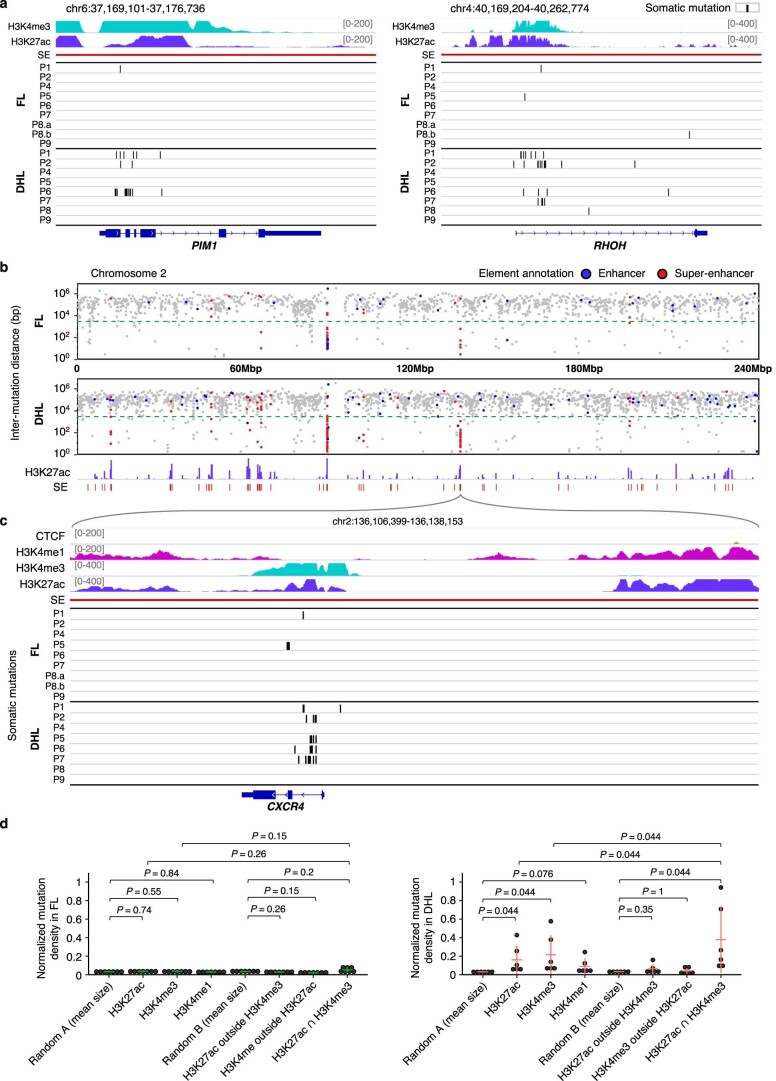
Examples of recurrent foci of transformation-associated aSHM. **(a-c)** Examples of recurrent foci of transformation-associated aberrant somatic hypermutation (aSHM), including *PIM1* and *RHOH* genes **(a)**, and aSHM occurring in the first intron of the *CXCR4* gene, shown on the rainfall plot in **(b)** and in relation to H3K4me3 and H3K27ac peaks in **(c)**. **(d)** Statistical evaluation of mutation density at various histone marks and equal size random areas for follicular lymphoma (left, n = 7 samples) and DHL (right, n = 6 samples). Each dot represents normalized mutation density in a specific genomic region for each tumor sample. The horizontal line in each group represents its mean value, and the corresponding vertical line represents its standard deviation bar. The group ‘Random A’ is composed of a series of randomly generated genomic regions with each single region size and total region size equal to the average single size and total size of all H3K27ac, H3K4me3, and H3K4me1 peaks respectively. The group ‘Random B’ is composed of a series of randomly generated genomic regions with each single region size and total region size equal to the average single size and total size of all H3K27ac outside H3K4me3, H3K4me3 outside H3K27ac, and H3K4me3 overlapping H3K27ac peaks respectively. *P*-values in each figure were calculated using two-sided Wilcoxon signed rank tests paired by tumor samples with false discovery rate (FDR) correction. Source data

Super-enhancers (SEs) are regulatory regions that often control the expression of lineage-specific genes to activate rapid transcription during cell differentiation. Many important B cell lineage-defining genes such as *AID*, *RAG1* and *PAX5* are regulated by neighboring SE sequences^35^. aSHM within SE clusters has been observed in human lymphomas and mouse B cells^10,11,13,35,36^; however, the evolution of SE mutations during lymphoma progression, their recurrent occurrence at SE-embedded promoters (in contrast to some reports that they occur surrounding enhancers) and their effects on gene expression in lymphoma cells are incompletely understood. Evaluation of FL/DHL pairs demonstrates accumulation of mutation clusters with short intermutational distances. These mutational signatures are similar to the kataegis mutagenesis seen in tumors due to the mutator activity of the AID/APOBEC family of proteins (Fig. 4a)^13,37,38^. A substantial number of mutations acquired upon transformation to DHL (11.74%) are geographically clustered at H3K27ac-enriched sites in the B cell genome (Fig. 4a). The enrichment of H3K27ac marks defines these sites as enhancer/SEs. Many of the genes we find to have transformation-associated enhancer/SE mutations, such as *BCL6*, *CIITA*, *IRF8* and *ZFP36L1*, have well-known mechanistic roles in B cell development and lymphomagenesis (Fig. 4b). The canonical immunoglobulin targets of SHM (*IGH*, *IGL* and *IGK*) also continue to acquire mutations during transformation to DHL (Fig. 4a,b). Transformation-associated noncoding mutations in the *CIITA* and *IRF8* genes are embedded in H3K27ac-enriched regions of the genome and adjacent to the TSS in the first intron of both genes (Fig. 4c), and observed recurrently in 5/8 and 6/8 DHL, respectively. Other examples of transformation-associated point mutations overlapping an SE occur at the *PIM1*, *RHOH* and *CXCR4* loci in 3/8, 4/8 and 5/8 tumors, respectively (Extended Data Fig. 2a–c). Noncoding RNAs, including miR-142, are also found to be recurrently mutated upon transformation to DHL (4/8 cases; Supplementary Fig. 2a–c).

**Fig. 5:**
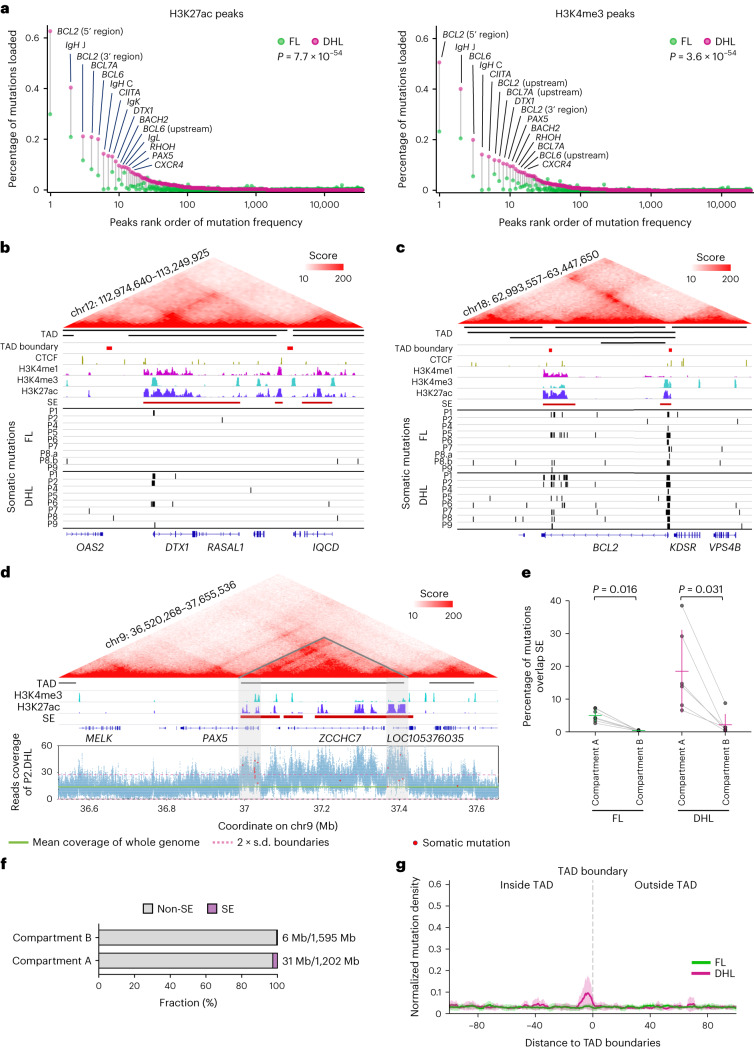
Enrichment of transformation-specific mutations at boundaries of topologically associating domains. **a**, Plots demonstrating percentages of mutations overlapping H3K27ac peaks (left, *n* = 35,770) and H3K4me3 peaks (right, *n* = 25,740) in DHL compared to FL. Each pair of points represents an H3K4me3/H3K27ac peak region. The 15 most frequently mutated peaks are highlighted and labeled with the names of nearby genes. The *P* values were calculated by two-sided Wilcoxon signed-rank test. **b**,**c**, In *DTX1* (**b**) and *BCL2* (**c**) transformation-associated aSHM overlaps H3K4me3 and H3K27ac sites and occurs near the boundaries of TADs. **d**, In the *PAX5*/*ZCCHC7* region, transformation-associated aSHM overlaps H3K4me3 and H3K27ac sites and occurs near the boundaries of the TAD. The increased read depth of the *PAX5*/*ZCCHC7* regions in P2.DHL is shown. **e**, Occurrence of SE mutations in compartment A versus compartment B in FL (left, *n* = 7) and DHL (right, *n* = 6) tumors with each pair of dots connected by a gray line representing one lymphoma sample. The *P* values were calculated using two-sided Wilcoxon signed-rank tests. The horizontal line in each group represents its mean value, and the corresponding vertical line represents its s.d. bar. **f**, Fraction of SE regions in compartment A versus compartment B. The bar plot shows size fraction of SE and non-SE in compartment A and compartment B, respectively. The overall total size of SEs across the whole genome is approximately 37 Mb; 31 Mb are located within compartment A and represent 2.6% of the overall compartment A region. **g**, Normalized and averaged somatic mutation density of FL and DHL in relation to TAD boundaries. Starting from the center of every TAD boundary (gray dashed line), the left side represents regions inside of TADs and the right side represents areas outside of TADs. Intra-TAD boundaries are not considered in this analysis. Source data

**Extended Data Fig. 3:**
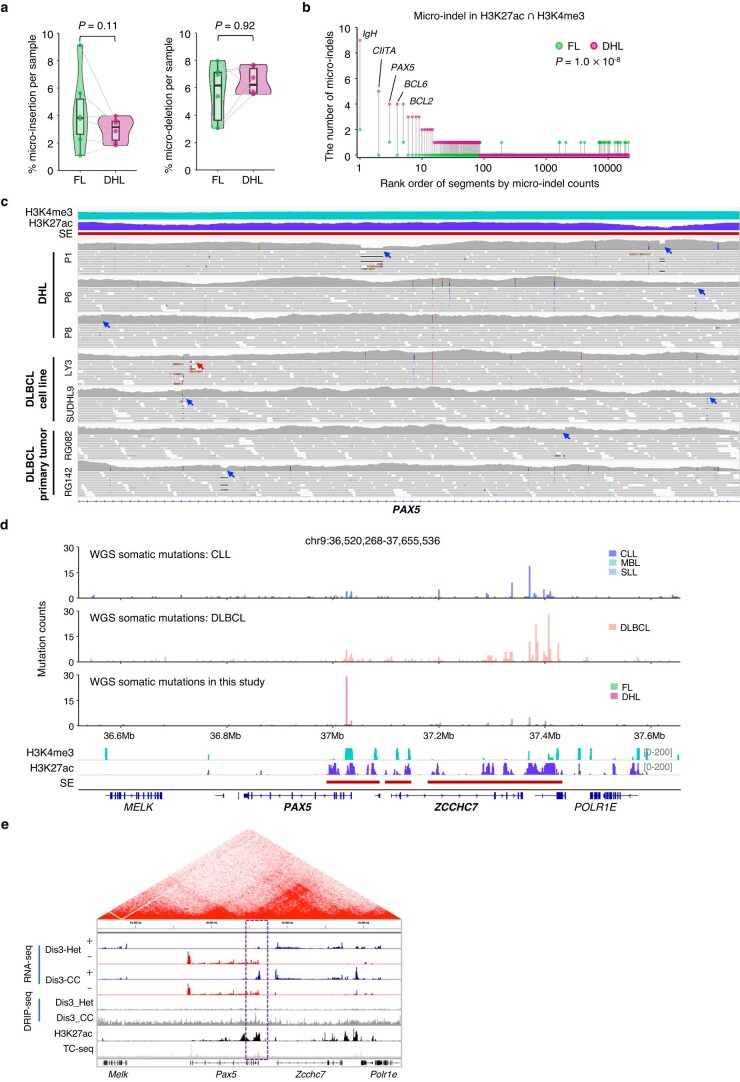
Micro-insertions and microdeletions observed in follicular lymphoma and double hit lymphoma, *PAX5*/*ZCCHC7* mutational hotspot in lymphoma, and the *PAX5*/*ZCCHC7* locus in mice. **(a)** Overall frequency of micro-insertions (left) and micro-deletions (right) observed in paired follicular lymphoma and DHL samples from n = 6 patients. Each pair of dots connected by a grey line represents a pair of follicular lymphoma and DHL samples from a single patient. The mutational frequencies on the y-axis are expressed as percentage of micro-insertions or micro-deletions out of all mutations. The *P*-values were calculated by two-sided Wilcoxon signed rank tests. The boxplots display the 25^th^ and 75^th^ percentiles and the median of each group of data. The whiskers represent the highest and lowest values within 1.5 ${\rm{\times }}$ the inter-quartile range. **(b)** The number of micro-indels overlapping H3K4me3 and H3K27ac intersecting regions in DHL compared to follicular lymphoma. Each pair of points represents an intersection segment of H3K4me3 peak and H3K27ac peak. All n = 21,283 segments were ranked in descending order by the sum of micro-indels observed in DHL. Five segments that harbor over three micro-indels are highlighted and labeled with nearby genes. The *P*-value was calculated by two-sided Wilcoxon signed-rank test. **(c)** Microdeletions and micro-insertions observed in the *PAX5*-TSS2 region in three DHL tumors, two diffuse large B cell lymphoma (DLBCL) cell lines, as well as two primary DLBCL tumors. In IGV, WGS data from these seven samples show eight micro-deletions (blue arrows) and one micro-insertion (red arrow) in this region. **(d)** Mutational hotspots in the *PAX5*/*ZCCHC7* locus previously reported in chronic lymphocytic leukemia (CLL)^43^ (top panel), DLBCL^34^ (middle panel), and in the current DHL series (bottom panel). All *p*-values were calculated by two-sided Wilcoxon signed-rank test. **(e)** Hi-C overlap of the *PAX5*/*ZCCHC7* locus shows bidirectional transcription (*Dis3*^*C*/*C*^ represents cells depleted of RNA exosome activity to stabilize bidirectional ncRNA expression), accumulation of non-B DNA associated DNA/RNA hybrids (in *Dis3*^*C*/*C*^ cells via DRIP-seq), H3K27ac peaks and AID target site identification via translocation capture sequencing (via HTGTS-seq). These observations demonstrate that processes relating to DNA targeting by AID occur at the *PAX5* promoter in mouse B cells. Source data

SEs contain both regulatory DNA sequences (enhancers) and promoters of genes^10,39^. We find that transformation-associated aSHM occurs predominantly at promoters within SEs. We find that aSHM is distributed within wide regions covered by H3K27ac marks (representing enhancers) and H3K4me1 marks (representing poised enhancers) and relatively more tightly around promoter marks (H3K4me3; Fig. 4d). Comparative analyses of mutation density in H3K27ac regions outside of H3K4me3 areas, H3K4me3 regions outside of those with H3K27ac marks and in areas representing the intersection of H3K27ac marks and H3K4me3 marks suggest that sequences surrounding or overlapping promoters embedded in H3K27ac-marked SEs are most frequently mutated during lymphoma progression (Fig. 4d and Extended Data Fig. 2d). Furthermore, the percentage of mutations overlapping H3K4me3 and H3K27ac regions increases at many important loci, including *BCL2*, *IGH*, *BCL6*, *CIITA*, *BCL7A*, *DTX1* and *PAX5*/*ZCCHC7*, upon transformation of FL to DHL (Fig. 5a). Because topologically associating domains (TADs) contain both SEs and their target genes^40^, and because loop extrusion and genome architecture-related proteins are implicated in aSHM^35^, we investigated whether SE-resident aSHM clusters reside within TADs. At many loci, we find lymphoma transformation-associated aSHM cluster around boundaries of TADs containing known aSHM targets including *DTX1* (Fig. 5b), *BCL2* (Fig. 5c) and *PAX5* (Fig. 5d). Genome-wide analysis of SE-associated aSHM locations shows that a high proportion of SE-associated aSHM observed upon transformation to DHL is located in compartment A, the spatial region of the genome containing open and active chromatin^41^ (Fig. 5e), consistent with the fact that a higher fraction of SEs is located in compartment A (Fig. 5f). aSHM tends to accumulate close to TAD boundaries where cohesin and CTCF proteins localize (Fig. 5g). Micro-insertions (4% of all somatic mutations) and microdeletions (6% of all somatic mutations) occur more frequently in H3K27ac-marked and H3K4me3-marked intersecting regions in DHL than in FL. The most frequent of these alterations occur at SEs of known aSHM targets, such as *IGH*, *CIITA*, *PAX5*, *BCL6* and *BCL2* (Extended Data Fig. 3a,b).

#### Different types of genetic alterations occur at the same locus

A large set of recurrently acquired copy number gains as well as some recurrently acquired losses (Fig. 3a,b), including loss of *CDKN2A*, which has been previously implicated in transformation of FL and other types of lymphomas^24,42^, occurs in the course of transformation to DHL. The recurrent transformation-associated copy number gain at the *PAX5*/*ZCCHC7* region extends from the 5′ region of the *PAX5* gene to the 3′ end of the neighboring *ZCCHC7* gene. Additionally, several transformation-associated mutations, representing an aSHM hotspot, occur near the promoter of the *PAX5* gene (Fig. 3c), overlapping an H3K4me3 peak and inside an H3K27ac cluster. Analyses of whole-exome sequencing and WGS data from other series of DLBCL^34^ and chronic lymphocytic leukemia^43^ also show mutations in the same region of the *PAX5*/*ZCCHC7* locus, providing further evidence of recurrent aSHM events in this region in B-NHL (Extended Data Fig. 3c,d).

**Fig. 6:**
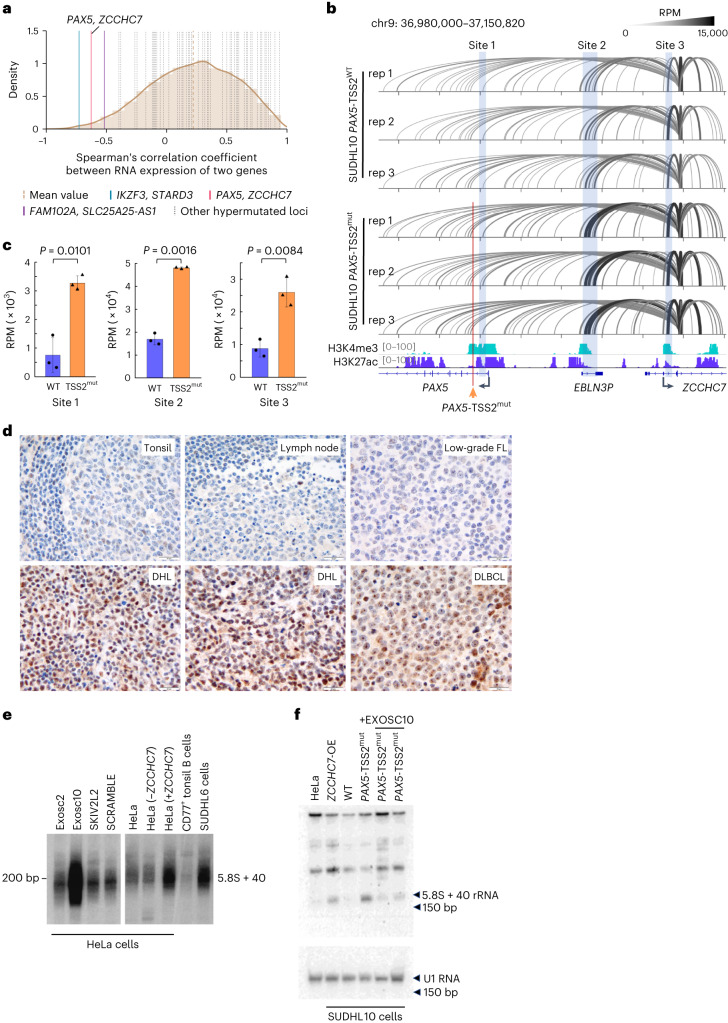
Overexpression of ZCCHC7 in large B cell lymphomas by enhancer retargeting causes ribosome RNA processing defects. **a**, Distribution of Spearman’s correlation coefficient between RNA expression of two genes located within the same TAD. In total, 19,676 gene pairs were investigated by analyzing transcriptomic data of 11 DLBCL cell lines to identify candidates of ER. The *ZCCHC7–PAX5* pair is highlighted. **b**, The 4C-seq experiments from the *ZCCHC7* promoter measuring the interaction with H3K27ac^+^ H3K4me3^+^ peaks, including those at the *PAX5* promoter. In SUDHL10 cells where the *PAX5*-TSS2 has been replaced with a mutation identified in DHL ‘chr9: 37,026,315–37,026,316 GC->AT’ (mutated region shown with orange arrow; SUDHL10 *PAX5*-TSS2^mut^), the *PAX5* intragenic enhancer regions (sites 1, 2 and 3) interact with the promoter of ZCCHC7 more strongly than in SUDHL10 cells without the *PAX5*-TSS2 mutation (see thickness of black lines). **c**, Normalized coverage of the 4C interactions for each 4C-seq viewpoint is shown. The TSS2 mutant region does not overlap the *ZCCHC7* interaction domain of the *PAX5* promoter. Data are presented as mean values ± s.d. Two-sided Welch’s *t* test was used; *n* = 3 for each group. **d**, Tissue microarrays containing triplicate samples of 33 DLBCL and 9 DHL with controls including benign spleen, lymph node, tonsil and low-grade FL were stained by immunohistochemistry for ZCCHC7. ZCCHC7 was visualized predominantly in nucleoli and sometimes in the nuclei. Scale bar, 20 µm. Examples of stained tumors and controls are shown. **e**, The DHL cell line SUDHL6 was separated on denaturing acrylamide gels and analyzed by northern blotting. CD77^+^ tonsillar B cells were used as controls. As additional controls, and to unambiguously map the various 5′ end extended forms of 5.8S, HeLa cells depleted of RNA exosome subunits (EXOSC2 and EXOSC10) or of a cofactor (SKIV2L2) are used (left). HeLa cells depleted of (− lane), or overexpressing (+ lane), ZCCHC7 were also included. Results represent two or more independently performed biological replicates. **f**, Total RNA extracted from HeLa cells, SUDHL10 cells overexpressing *ZCCHC7* (SUDHL10-OE), parental SUDHL10 (WT), *PAX5*-TSS2^mut^ (SUDHL10 cells with *PAX5* promoter DHL mutation) and two sets of *PAX5*-TSS2^mut^ transfected with Exosc10 were processed as in **e**. Equal loading is shown with the U1 RNA control (bottom). Results represent two independently performed experiments. RPM, reads per million. Source data

**Extended Data Fig. 4:**
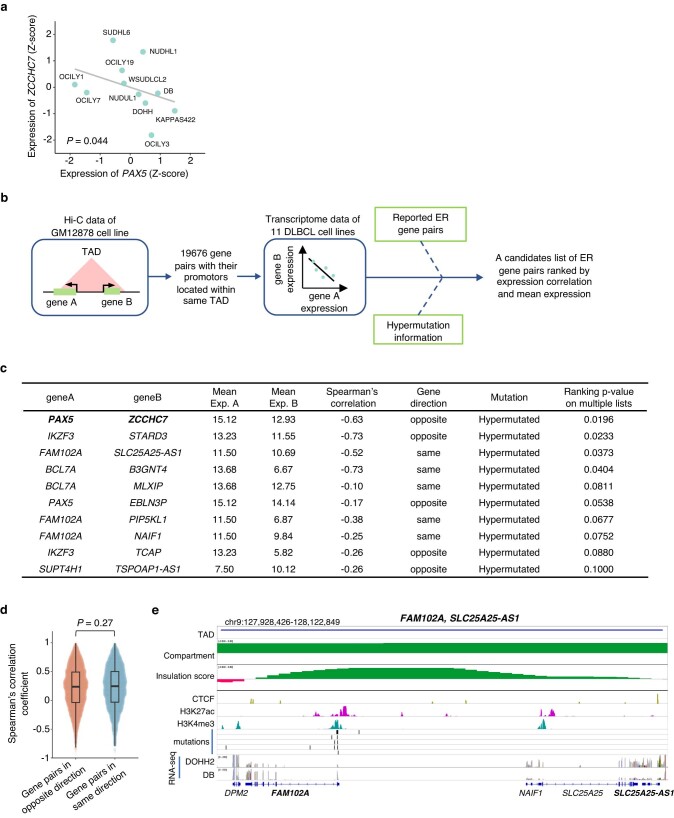
Prediction of enhancer retargeting. **(a)** Significant negative correlation between RNA expression of *PAX5* and *ZCCHC7* in 11 DLBCL cell lines. The *P*-value was calculated by two-sided Spearman’s correlation test. **(b)** Schematic of the pipeline used to identify potential targets of enhancer retargeting in the B cell lymphoma genome. Overall results of this analysis are shown in Fig. 4b. The transcriptomic data from 11 DLBCL cell lines were reported by Morin et al.^34^. The list of 367 enhancer retargeting (ER) gene pairs was previously reported by Oh et al.^44^. **(c)** Top 10 potential enhancer retargeting gene pair candidates ranked by the pipeline and filtered by ‘Hypermutated’ criteria. The P-values were estimated for ranking consistently high (or low) on multiple lists (see Methods). **(d)** Comparison of Spearman’s correlation coefficient between predicted gene pairs experiencing enhancer retargeting and affected in opposite directions (n = 10,158) versus those affected in the same direction (n = 9,518). The *P*-value is calculated by two-sided Wilcoxon rank sum test. The boxplots display the 25^th^ and 75^th^ percentiles and the median of each group of data. The whiskers represent the highest and lowest values within 1.5 $\times$ the inter-quartile range. **(e)** Proximity of *FAM102A* and *SLC25A25-AS1* and somatic mutations occurring in the promotor of *FAM102A*. Source data

**Extended Data Fig. 5:**
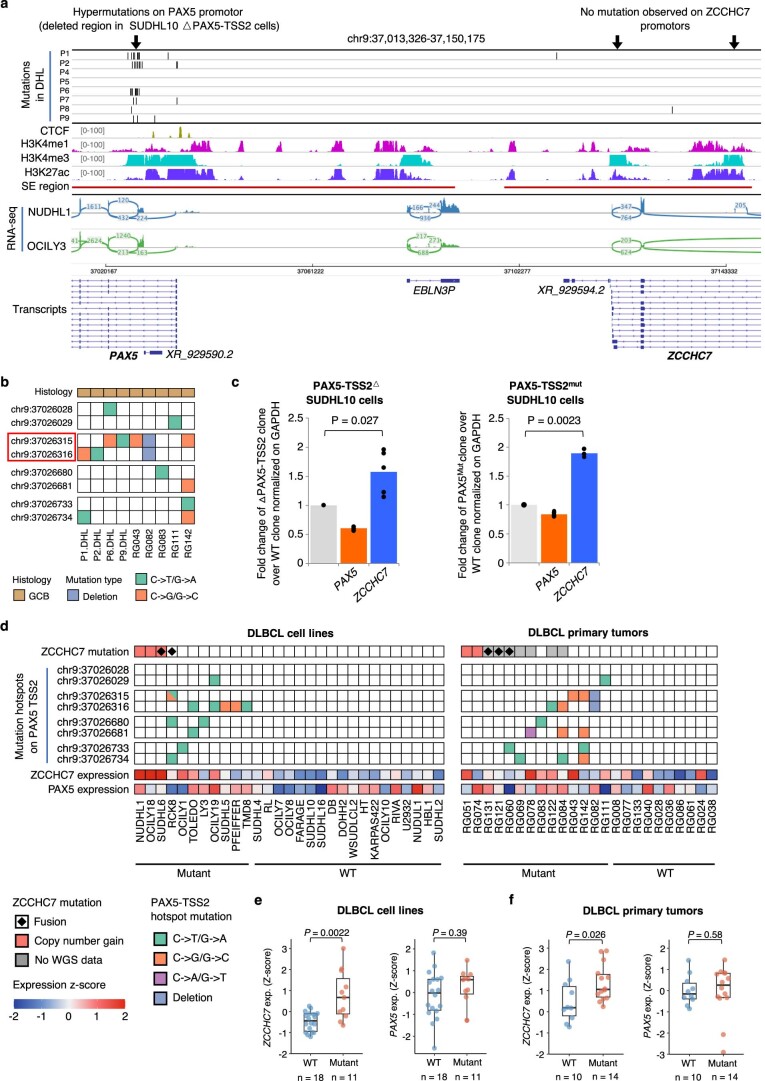
Overexpression of *ZCCHC7* in DLBCL cell lines and primary tumors that harbor *ZCCHC7* fusion, *ZCCHC7* copy number gain, or *PAX5*-TSS2 mutation. **(a)** Transcript reconstitution of *PAX5* demonstrates two TSS, canonical TSS and *PAX5*-TSS2. The distribution of H3K4me3, H3K27ac, and aSHM overlapping the two TSS is shown. **(b)** *PAX5*-TSS2 hotspot mutations and deletions seen in four of the DHLs in this study (P1, P2, P6, P9) and in other studies^34^. The most frequent mutations, that characterize the *PAX5*-TSS2^mut^ in Fig. 6 are marked by a red frame. **(c)** In the SUDHL10 cells, deletion of *PAX5*-TSS2 (left) or incorporation of TSS2^mut^ (right) leads to marginally decreased expression of *PAX5* mRNA but significantly increased expression of *ZCCHC7*. Student’s 2-tailed T-test (paired) used for statistical analysis in both panels. **(d)** Mutation heatmap of 29 DLBCL cell lines (left panel) and 24 DLBCL primary tumors (right panel) at *ZCCHC7* and *PAX5* TSS2. Cell line WGS/RNAseq data were obtained from CCLE and SRA: PRJNA854968. Tumor WGS/RNAseq data were obtained from dbGaP: phs000235.v20.p6. Cell lines or tumors showing *ZCCHC7* copy number gain, *ZCCHC7* fusion, or mutation in any of four *PAX5*-TSS2 hotspots (as shown in **(b)**) are included in the ‘Mutant’ group. Others, displaying no alteration in these regions, compose the ‘WT’ group. All of the *ZCCHC7* fusion events were detected via WGS or RNAseq data and summarized in Extended Fig. 7. All of the hotspot mutations were confirmed by checking corresponding WGS data or RNAseq data in IGV, with at least five supporting reads. Six primary tumors without WGS data but showing *ZCCHC7* fusion or *PAX5*-TSS2 hotspot mutation in RNAseq data were also included. ‘WT’ samples showing poor read coverage (depth < 10) at the four mutational hotspots were discarded. Z-score transformed RNA expression of *ZCCHC7* and *PAX5* are shown at the bottom. **(e and f)** RNA expression of ZCCHC7 and PAX5 in the ‘WT’ and ‘Mutant’ groups of DLBCL cell lines (**e**) and tumors (**f**) described in (**d**). P-values were calculated by two-sided Wilcoxon rank-sum test. Boxplots display the 25th and 75th percentiles and median of each group. Whiskers represent the highest and lowest values within 1.5 ${\rm{\times }}$ the inter-quartile range. Source data

**Extended Data Fig. 6:**
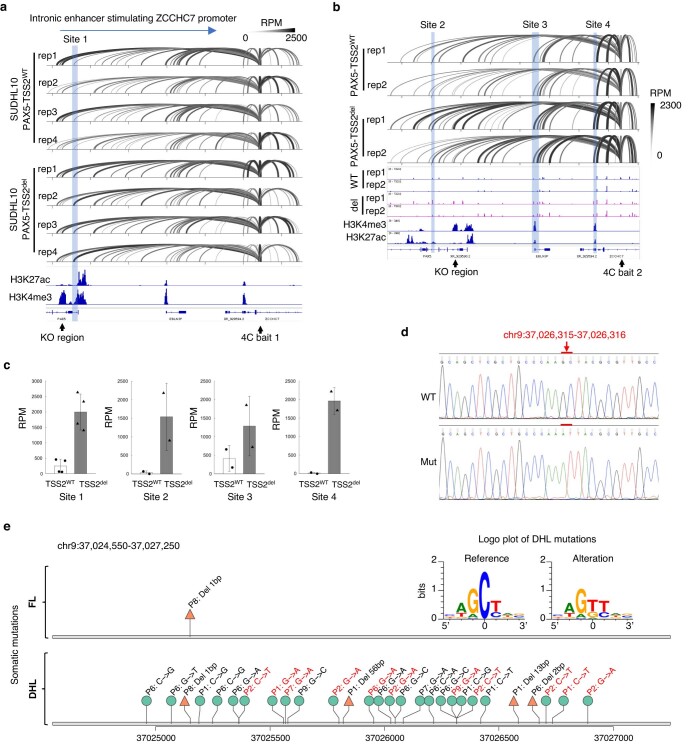
Enhancer retargeting at the *PAX5*/*ZCCHC7* locus resulting from aSHM at the *PAX5* alternative promoter/TSS. **(a)** Four independent 4C-seq experiments measuring interactions between the *ZCCHC7* promoter and intragenic enhancer regions adjacent to *PAX5*. In ***Δ****PAX5*-TSS2 SUDHL10 (*PAX5*-TSS2 deletion shown with black arrow), the *PAX5* intragenic enhancer region interacts more strongly with the promoter of *ZCCHC7* relative to cells with intact *PAX5*-TSS2 (see thickness of black lines). **(b)** Comparison of 4C-seq data from *PAX5* TSS2^WT^ versus *PAX5* TSS2^del^ cells, demonstrating that similar regions of the *ZCCHC7-PAX5* SE show increased interaction with the *ZCCHC7* gene. **(c)** The normalized coverage of the interactions (site 1, site 2, site 3, and site 4) in **(a and b)** is shown in the bar graph. Error bars represent the standard deviations. Two-sided Welch’s t-test were used; n = 4 for interaction between site 1 and 4C bait 1, n = 2 for other interactions. **(d)** Sanger sequencing of chr9:37,026,299-37,026,327 in SUDHL10 *PAX5*TSS^mut^ cells. The two mutations are located at chr9:37,026,315-37,026,316 (as in Extended Data Fig. 5b). **(e)** Summary of *PAX5* H3K4me3 region mutations acquired upon transformation to DHL. The weblogos show the classical AID mutational signature, C→T/G→A with WRC/GYW motif (W = A/T, R = A/G, and Y = T/C), that dominates the DHL-specific mutations within this 3kb region of *PAX5*. Specifically, 11 C→T/G→A mutations out of 28 DHL-associated mutations in this region reflect the WRC/GYW motif (labeled in red). Source data

We next sought to understand how aSHM at *PAX5*/*ZCCHC7* affects expression of one or both genes in DHL. A common set of enhancers has been found to control the expression of both *ZCCHC7* and *PAX5* (refs. ^44,45^), and we suspected that ‘enhancer retargeting (ER),’ whereby functional loss of a promoter results in subsequent preferential targeting of a different promoter^35,45^, could occur following aSHM at promoter regions during lymphoma progression. Supporting this possibility, we observe that *PAX5* and *ZCCHC7* gene promoters exist in the same TAD in mice (Extended Data Fig. 3e) and humans (Fig. 5d)^35^ and that *PAX5* and *ZCCHC7* expression is negatively correlated in DLBCL cell lines (Extended Data Fig. 4a). Bioinformatic prediction of ER was carried out using genomes of the 8 DHLs in our study and 39 published DLBCLs^34^ (Extended Data Fig. 4b). In total, 143 gene pairs from 25 hypermutated loci were investigated. We identified several gene pairs that might undergo ER, including the *PAX5-ZCCHC7* locus (Fig. 6a), *IKZF3-STARD*3 locus, *FAM102A-SLC25A25-AS1*, *BCL7A-B3GNT4* and others (Extended Data Fig. 4c–e). To evaluate potential ER in the *PAX5*/*ZCCHC7* locus, we deleted the *PAX5* promoter alternate TSS (*PAX5*-TSS2), which was found to be mutated in our DHL cohort (Extended Data Fig. 5a,b) in the SUDHL10 cell line, using CRISPR/Cas9 homology-directed repair/mutagenesis (HDR), and observed a resulting increase in *ZCCHC7* mRNA expression (Extended Data Fig. 5c, left). Additionally, several DLBCL cell lines and primary human DLBCLs harbor *PAX5*-TSS2 mutations (Extended Data Fig. 5d and Supplementary Table 4) and on average demonstrate increased *ZCCHC7* mRNA levels relative to cell lines and tumors without such mutations (Extended Data Fig. 5e,f), suggesting that *PAX5* promoter mutations might promote *ZCCHC7* overexpression in lymphoma cells. In 4C assays using two baits within the *ZCCHC7* locus, the enhancer regions located close to *PAX5-*TSS2 (Extended Data Fig. 6a) and within the *PAX5*/*ZCCHC7* SE (sites 2, 3 and 4) show stronger interaction with the *ZCCHC7* gene following deletion of the *PAX5-*TSS2 (Δ*PAX5*-TSS2) region (Extended Data Fig. 6a–c). Next, we incorporated the recurrent *PAX5*-TSS2 mutations (Chr9:37,026,299–37,026,327:GC to AT conversion labeled as *PAX5*-TSS2^mut^; Extended Data Fig. 6d) into the SUDHL10 cell line using homology-directed genome editing^46^ and found that the enhancer regions (sites 1, 2 and 3) in the *PAX5-ZCCHC7* SE cluster interact more efficiently with the *ZCCHC7* promoter (Fig. 6b,c), compared to unmutated cells. Comparison of 4C assays performed with Δ*PAX5*-TSS2 and *PAX5*-TSS2^mut^ identified overlapping interaction regions in the *PAX5-ZCCHC7* SE that loop to the *ZCCHC7* promoter. Given that both deletion and point mutation of *PAX5-*TSS2 lead to stronger interactions between the *PAX5* enhancer region and the *ZCCHC7* promoter and a corresponding increase in *ZCCHC7* mRNA expression (Extended Data Fig. 5c), we postulate that *PAX5* promoter mutations in lymphoma cells increase *ZCCHC7* expression. Notably, this mechanism could explain the *ZCCHC7* overexpression that we observe in lymphomas that do not harbor *PAX5*/*ZCCHC7* copy number gains but have *PAX5-ZCCHC7* SE mutations. Consistently, we found several DLBCL cell lines and primary human DLBCLs to harbor *PAX5-*TSS2 mutations (Extended Data Fig. 5d).

**Extended Data Fig. 7:**
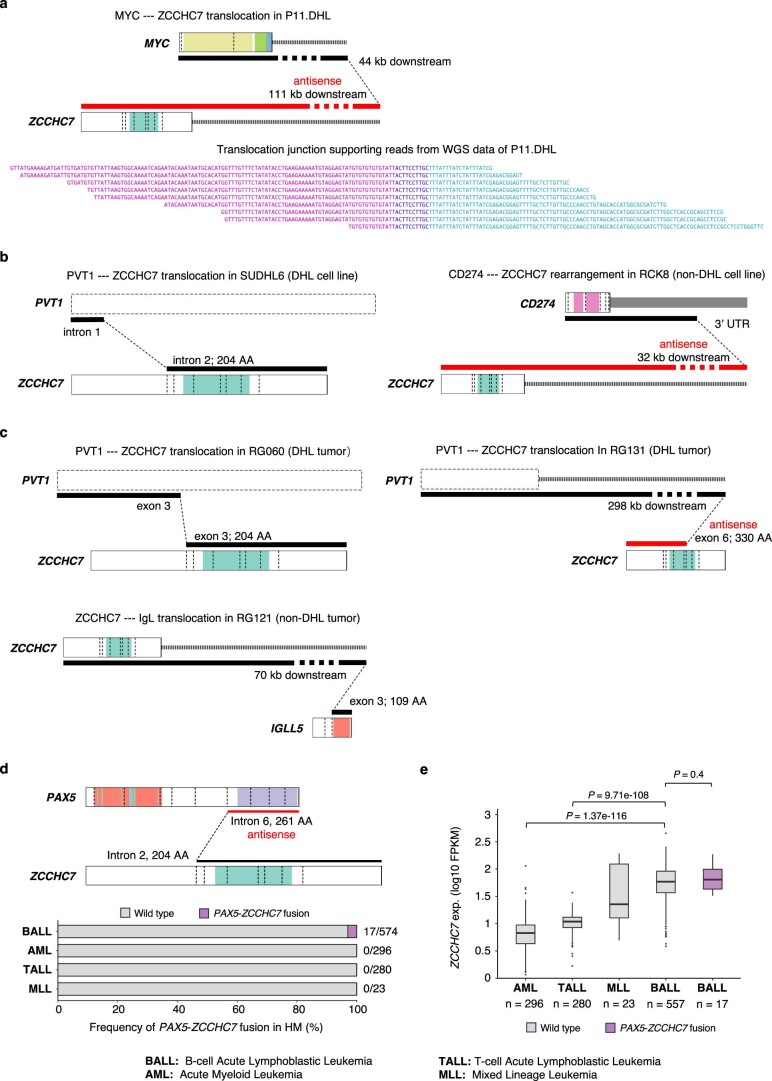
Structural variants involving *ZCCHC7*. **(a)** *MYC-ZCCHC7* translocation identified in the DHL from P11. P11 represents a de novo DHL (no prior follicular lymphoma) selected for sequencing due to a high degree of ZCCHC7 expression observed on IHC, but not included in the main cohort of paired longitudinal samples. The translocation junction is observed between *MYC* downstream and *ZCCHC7* downstream. **(b)** *PVT1-ZCCHC7* translocation and *CD274-ZCCHC7* rearrangement identified in SUDHL6 and RCK8 cell lines, respectively. The breakpoints in SUDHL6 cells were observed within the two genes, while the RING Zn-finger domain (light green) of *ZCCHC7* remained in the fused transcript. Break points of the *CD274-ZCCHC7* fusion in the RCK8 cell line are located in the 3′ UTR of *CD274* and 32 kb downstream of *ZCCHC7*. The fusion-supporting junction reads display *ZCCHC7* in an antisense direction to *CD274*. **(c)** Three *ZCCHC7* translocations detected in RNAseq data from primary DLBCL tumors reported by Morin et al.^34^. The *PVT1-ZCCHC7* translocations were observed in two DHL patients. The majority of the functional domain (teal) of *ZCCHC7* is preserved in the two fusion transcripts. The *ZCCHC7-IGL* translocation, which joined downstream of *ZCCHC7* and *IGLL5* exon 3, was detected in a DLBCL. All the RNAseq data supported gene fusions were detected by Arriba 2.3.0 and checked in IGV. **(d)** Investigation of the occurrence of the *PAX5-ZCCHC7* gene fusion among n = 1,173 hematopoietic malignancies (HM) samples reported in the St. Jude Cloud ProteinPaint portal. The *PAX5-ZCCHC7* gene fusions that connect intron 6 of *PAX5* and intron 2 of *ZCCHC7* in an antisense direction were reported in 17 cases of B-ALL. **(e)** Comparison of *ZCCHC7* expression among four types of HM. B-ALL and B-ALL with known *PAX5-ZCCHC7* fusions show the highest expression levels of *ZCCHC7* compared to other types. The *P*-values were calculated using two-sided Wilcoxon rank-sum tests. The boxplots display the 25^th^ and 75^th^ percentiles and the median of each group of data. The whiskers represent the highest and lowest values within 1.5 $\times$ the inter-quartile range. Source data

As in humans, the mouse *Zcchc7* gene resides within a TAD (Extended Data Fig. 3e). Translocation capture sequencing experiments identify AID-induced translocations overlapping the *Pax5* promoter, similar to our observations in the human *PAX5*-TSS2, showing that this sequence accumulates AID-mediated genomic alterations in both human and mouse genomes. Furthermore, RNA-seq experiments performed in mouse B cells show sense/antisense transcription overlapping the *Pax5* promoter region and deletion of RNA exosome activity (*DIS3*^*C*/*C*^ B cells) leading to accumulation of these sense/antisense transcripts, a mechanism resulting in increased AID-mediated mutagenesis^12,47,48^. DNA/RNA hybrid immunoprecipitation followed by high-throughput sequencing demonstrates accumulation of R-loops at this site, and therefore generation of AID substrates. H3K27ac chromatin immunoprecipitation followed by sequencing (ChIP–seq) identifies the overlapping SE region (Extended Data Fig. 3e). In summary, as AID-mediated SHM occurs within SEs that are marked with overlapping sense/antisense transcription, and preferentially targets non-B DNA regions^5,10–14^, our findings support AID-mediated aSHM at the *PAX5* promoter sequence. Many of the *PAX5* promoter mutations observed in our cohort demonstrate the classical AID mutational signature (Extended Data Fig. 6d)^49^. Furthermore, sequencing of DHL samples has shown that *ZCCHC7* represents a frequent translocation partner of *MYC*^50,51^ (such a translocation was also identified in one of our DHL samples separate from the longitudinal cohort, patient 11; Extended Data Fig. 7a). Additionally, in individual cell lines and tumors, *ZCCHC7* translocates with *PVT1* (Extended Data Fig. 7b,c), underscoring the susceptibility of this locus to AID-mediated genomic alterations.

*PAX5-ZCCHC7* structural variations are also observed in a subset of B-lymphoblastic leukemia (B-ALL), a neoplasm that demonstrates robust ZCCHC7 expression overall (Extended Data Fig. 7d), and especially in treatment-refractory cases, with *PAX5-ZCCHC7* positive cases showing the highest levels of *ZCCHC7* mRNA expression (Extended Data Fig. 7e). It is likely that in B-ALL this *PAX5-ZCCHC7* fusion is caused by a different mechanism driven by the RAG enzymes, although AID could also have a role^52,53^.

#### *PAX5*/*ZCCHC7* alterations affect pre-rRNA processing

**Extended Data Fig. 8:**
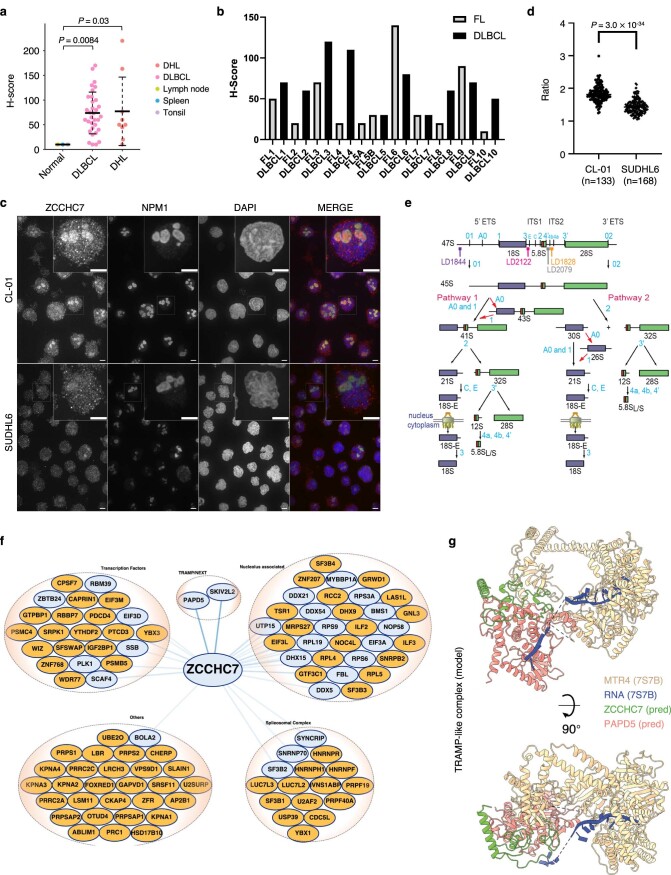
ZCCHC7 protein expression in lymphoma tissue, ZCCHC7 interaction network, reconstituted structure of the TRAMP complex using alpha-fold, and ZCCHC7 localization in SUDHL6 cells. **(a)** Tissue microarrays (TMAs) were immunohistochemically stained for ZCCHC7. DLBCL (N = 33, p = 0.0084) and DHL (N = 9, p = 0.03) showed a higher average degree of ZCCHC7 expression than individual benign lymph node, spleen, or tonsil, all of which showed a very low degree of ZCCHC7 expression. Mean H-score and standard deviation for each group are shown, with p-values calculated by two-sided Wilcoxon rank-sum test. **(b)** Longitudinal samples representing transformation of follicular lymphoma to DLBCL in 10 patients (separate from the sequenced cohort) were stained for ZCCHC7. ZCCHC7 expression usually increased upon lymphoma transformation. **(c)** Comparison of endogenous ZCCHC7 in the non-neoplastic human B cell line CL-01 and SUDHL6 (a DHL line that overexpresses ZCCHC7). NPM1 defines the nucleolus and DAPI counterstains nuclei (blue). ZCCHC7 accumulates in nucleoli of CL01 cells while also present in the nucleoplasm of SUDHL6 (scale bar 5 µm). **(d)** The ratio of nucleolar to total nuclear signal is significantly reduced in SUDHL6 compared to CL-01. Statistical significance was assessed using an unpaired two-tailed *t*-test, and the number of cells analyzed in each case is indicated. **(e)** Pre-rRNA processing pathway. Three out of four mature rRNAs, the 18S, 5.8S, and 28S are encoded as a long polycistronic precursor synthesized by RNA polymerase I, the 47S. The mature rRNA sequences are produced by extensive processing (cleavage sites indicated in blue). In the 47S, the mature rRNAs are interspersed by 5′ and 3′ external transcribed spacers (ETS) and internal transcribed spacers (ITS) 1 and 2. The probes used in northern blotting (LD1828, LD1844, LD2079, and LD2122) are highlighted. **(f)** FLAG-tagged ZCCHC7 protein and a FLAG-tag peptide were overexpressed in 293T cells to purify ZCCHC7 interacting factors via Mass spectrometry. The components of the RNA exosome complex essential cofactor NEXT directly interact with ZCCHC7, as does a set of nucleolar proteins. **(g)** Predicted structure model of the human TRAMP-like complex. The complex consists of the RNA helicase MTR4 (PDB ID: 7S7B, yellow), RNA (PDB ID: 7S7B, blue), the zinc-knuckle protein ZCCHC7 (AlphaFold prediction, green), and the noncanonical poly(A) polymerase PAPD5 (AlphaFold prediction, red). Ribbon representation of the model (left) and 90° rotation (right) highlighting binding of RNA and its recruitment to the RNA channel in MTR4. Source data

**Extended Data Fig. 9:**
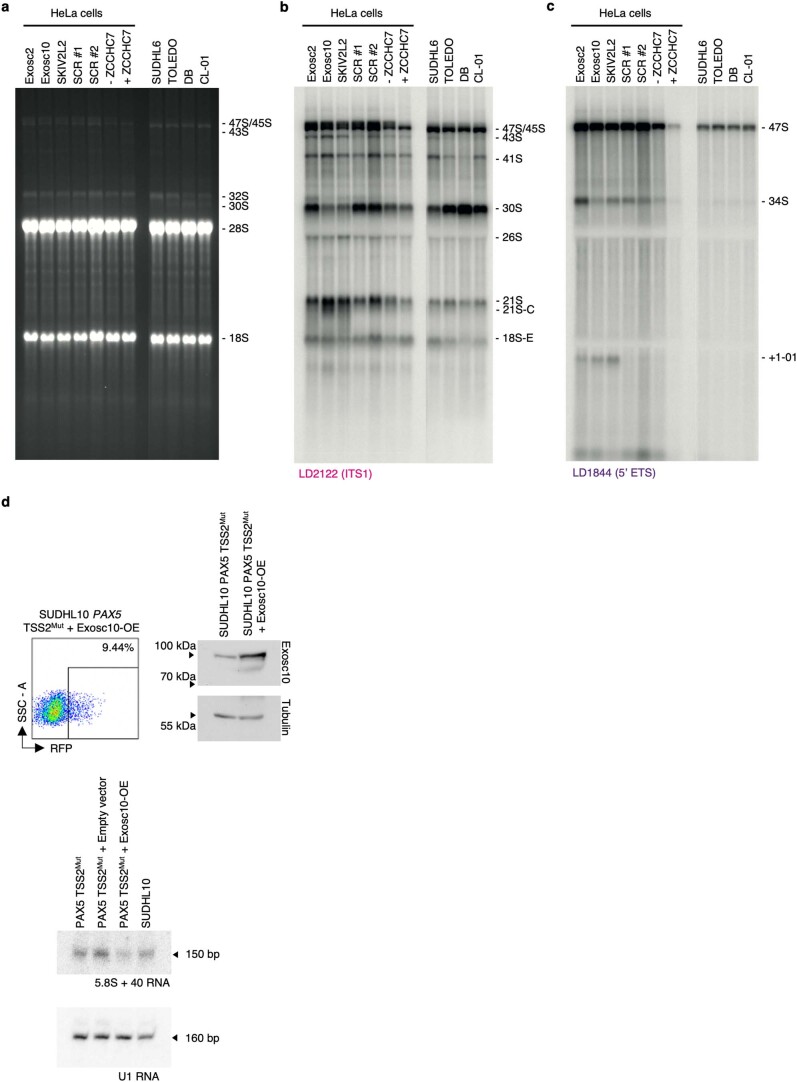
Evaluating the effect of ZCCHC7 overexpression on rRNA processing. **(a-c)** Total RNA extracted from the indicated cells was separated by denaturing agarose gel electrophoresis, the gel was stained with ethidium bromide (panel **a**), transferred to a nylon membrane and probed with radioactively-labeled anti-sense oligonucleotides specific to ITS1 **(b)** or 5′ ETS **(c)** SCR#1 is a non-targeting scrambled siRNA used to control HeLa depleted of EXOSC2, EXOSC10, and SKIV2L2. SCR#2 is non-targeting scrambled siRNA used to control HeLa depleted of ZCCHC7. The experiments represent results from 3 independently performed experiments. **(d)** Overexpression of *Exosc10* (*Exosc10*-OE) in *PAX5*-TSS2^mut^ SUDHL10 cells (*PAX5*-TSS2^mut^) rescues 5.8S + 40 rRNA processing. *PAX5*-TSS2^mut^ SUDHL10 cells were transfected with a control vector that does not contain *Exosc10* or with an *Exosc10* expression vector also containing green fluorescent protein (GFP) (See western blot showing Exosc10 expression) and cells were sorted by red fluorescent protein (RFP) expression. The flow cytometric experiment was repeated three times. Total RNA was isolated from GFP positive cells and separated on a 7% acrylamide gel, transferred to a membrane and blotted with a probe against 5.8S+40 rRNA. The level of 5.8S+40 rRNA decreases following *Exosc10* overexpression (lane 3), as compared to untransfected *PAX5-*TSS2^mut^ cells (lane 1), *PAX5*-TSS2^mut^ cells containing the control vector (lane 2), or unmutated and untransfected SUDHL10 cells (lane 4). Source data

To investigate the degree to which FL to DHL transformation alters *ZCCHC7* expression, we performed immunohistochemistry for ZCCHC7 using tissue microarrays (TMAs) containing 33 DLBCLs and 9 DHL samples and using pairs of lymphomas representing FL transformation to DLBCL from nine patients (these stained pairs were distinct from our sequenced DHL cohort; Extended Data Fig. 8a,b). We find that ZCCHC7 is more highly expressed in most DHL and DLBCL samples compared to benign lymphoid tissue and FL and that expression usually increases upon transformation to DLBCL (Fig. 6d and Extended Data Fig. 8a,b). ZCCHC7 is observed predominantly within the NPM1-stained nucleolus of human, nonneoplastic CL-01 B cells (Extended Data Fig. 8c), but this nucleolar localization is perturbed in SUDHL6 DHL cells that harbor a *ZCCHC7-MYC* translocation and show diffuse nuclear staining in addition to nucleolar staining (Extended Data Fig. 8c; nucleolar/nucleoplasmic ZCCHC7 distribution shown in Extended Data Fig. 8d). These observations raise the possibility of nucleolar dysregulation with changes in rRNA processing and ribosome biogenesis. The formation of the 3′ end of 5.8S rRNA is a complex multistep process involving sequential exoribonucleolytic digestion of internal transcribed spacer 2 (ITS2) by the RNA exosome (Extended Data Fig. 8e). Multiple subunits of the exosome are involved, with the trimmed precursors ‘handed over’ from one subunit to another, aided by cofactors^54–56^. The yeast Trf4/5-Air1/2-Mtr4 polyadenylation (TRAMP) complex participates in RNA degradation via interactions with the exosome^57^. ZCCHC7 potentially represents part of a human TRAMP-like complex, interacting with the noncanonical poly(A) polymerase, PAPD5 and the RNA helicase, MTR4 (Extended Data Fig. 8f) with RNA threading through the complex as observed in an AlphaFold^58^ reconstituted model (Extended Data Fig. 8g). We therefore hypothesized that ZCCHC7 could regulate the nucleolar function of the RNA exosome and also function independently in rRNA processing with Exosc10 (refs. ^55,59,60^). We were therefore interested to learn if pre-rRNA processing and ITS2 maturation, in particular, are altered in lymphoma cells upon ZCCHC7 overexpression. Total RNA extracted from DHL and control cells was analyzed by high-resolution northern blotting with probes detecting major 3′ extended precursor forms of 5.8S rRNA (Fig. 6e). The 5.8S + 40 is a normal pre-rRNA precursor, which is converted into 5.8S by the RNA exosome subunit EXOSC10 (ref. ^60^). The ZCCHC7-overexpressing SUDHL6 DHL cell line displays strikingly elevated levels of 5.8S + 40 relative to control CD77^+^ human tonsillar B cells (Fig. 6e, right). On the other hand, HeLa cells, used as an additional control, also do not show elevated levels of 5.8S + 40 (Fig. 6e, right). However, when ZCCHC7 is overexpressed in HeLa cells, 5.8S + 40 shows a marked increase similar to that observed in DHL cell lines (Fig. 6e). Specific silencers that deplete ZCCHC7 do not greatly affect 5.8S + 40 levels in HeLa cells (Fig. 6e, lane 2, right). The effect of ZCCHC7 overexpression on 5.8S + 40 rRNA processing in Hela cells or in SUDHL6 is specific to this step of rRNA processing as no major changes are observed at other steps of rRNA processing; in particular, production of mature 18S and 28S rRNA is not affected (Extended Data Fig. 9a–c). Thus, the overexpression of ZCCHC7 appears to particularly impact ITS2 processing, resulting in the accumulation of 5.8S + 40 rRNA. Next, we checked whether PAX5-TSS2^mut^ incorporated in SUDHL10 lymphoma cells, using Cas9-driven HDR, would cause 5.8S + 40 rRNA accumulation. We find that unmutated SUDHL10 cells have an inherent level of 5.8S + 40 rRNA, but as seen either following ZCCHC7 overexpression (Fig. 6f, lane 2) or following targeted incorporation of *PAX5*-TSS2 mutations (Fig. 6f, lane 4) there is a significant increase in the 5.8S + 40 rRNA level. Maturation of 5.8S + 40 rRNA occurs via the 3′ end RNA trimming activity of Exosc10, whose deletion also results in accumulation of 5.8S + 40 rRNA^60^ (Fig. 6e). Overexpression of Exosc10 in PAX5-TSS2^mut^ cells leads to rescue of 5.8S rRNA processing (Fig. 6f and Extended Data Fig. 9d). Taken together, ZCCHC7-mediated alterations of pre-rRNA processing kinetics may lead to production of a distinct ribosomal population, with repercussions for protein synthesis, efficiency and fidelity.

#### *PAX5*/*ZCCHC7* alterations affect nascent protein synthesis

**Fig. 7:**
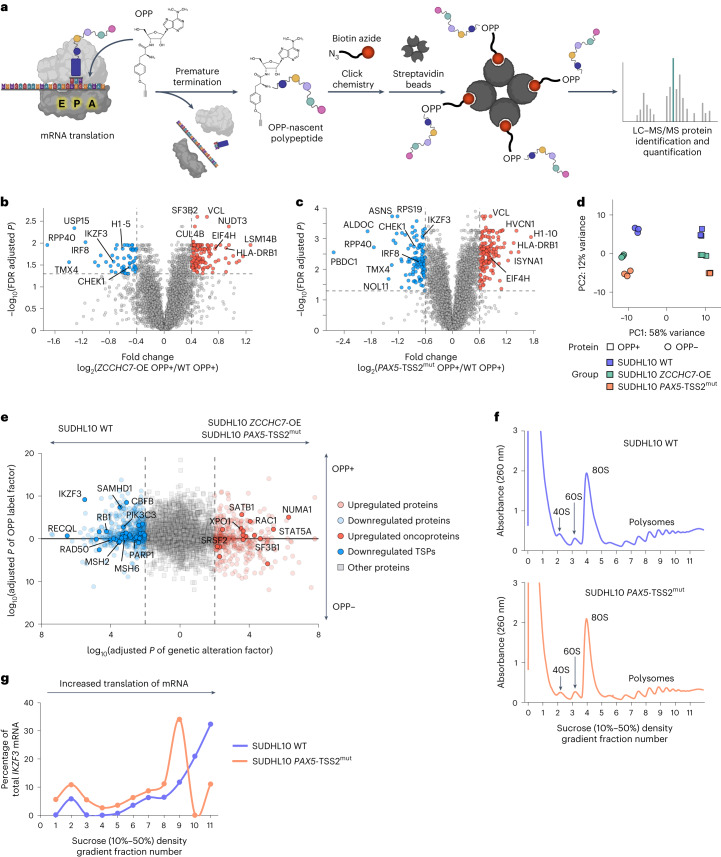
ZCCHC7 overexpression leads to dysregulation of protein synthesis. **a**, Schematic of nascent polypeptide synthesis analyses using OPP-ID^61^ (created with BioRender.com). **b**,**c**, Volcano plots showing differences in abundance of OPP+ proteins, in SUDHL10 cells with *ZCCHC7*-OE (**b**) or *PAX5*-TSS2^mut^ (**c**), compared to the SUDHL10 WT cells. The proteins that are significantly upregulated or downregulated in *ZCCHC7*-OE or *PAX5*-TSS2^mut^ are highlighted in red or blue, respectively. **d**, Principal component analysis plot of the proteomics data from SUDHL10 WT cells (no inherent mutation or alterations in *ZCCHC7* or *PAX5*-TSS2), *ZCCHC7*-OE and *PAX5*-TSS2^m^^ut^, with six biological replicates per group. Three replicates each were cultured with or without OPP (OPP+ and OPP−, respectively). Protein signals detected in the OPP+ group are shown as the union of OPP-labeled nascent proteins and nonspecifically captured background proteins. The OPP− data, representing protein signals from nonspecifically captured background proteins, were used as control to distinguish nascent proteins from background proteins. **e**, Two-way ANOVA test to evaluate the effect of genetic alteration (*ZCCHC7*-OE or *PAX5*-TSS2^m^^ut^) and OPP label on proteomic intensity. The plot shows FDR adjusted *P* values of the two factors (*x* axis for genetic alteration and *y* axis for OPP label) in the two-way ANOVA test. Each dot represents a protein and shows the abundance of corresponding proteins that decreased (left side) or increased (right side) in the *ZCCHC7*-OE and *PAX5*-TSS2^m^^ut^ cells. The upregulated/downregulated proteins were chosen by setting the adjusted genetic alteration *P* value < 0.01. Oncoproteins that increased and tumor suppressor proteins (TSPs) that decreased in SUDHL10 *ZCCHC7*-OE and SUDHL10 *PAX5*-TSS2^m^^ut^ cells are highlighted in red and blue, respectively. Two-sided Fisher’s exact tests were conducted to evaluate the relationship between oncoproteins/TSPs and upregulation/downregulation. The results show significant association of TSPs and downregulation with *P* = 0.0016. **f**,**g**, Polysome analyses of SUDHL10 cells and SUDHL10 *PAX5*-TSS2^m^^ut^ cells. The distribution of *IKZF3* mRNA (**g**) to heavier (highly translated fractions) decreases following *PAX5*-TSS2 mutation. Gross proteome-wide translational perturbation is not observed in *PAX5*-TSS2^m^^ut^ cells based on comparable polysome profiles (**f**), but specific mRNAs (for example, *IKZF3*) are translated differently (**g**). The data represent conclusions drawn from two independent biological replicates. ANOVA, analysis of variance. FDR, false discovery rate. Source data

**Extended Data Fig. 10:**
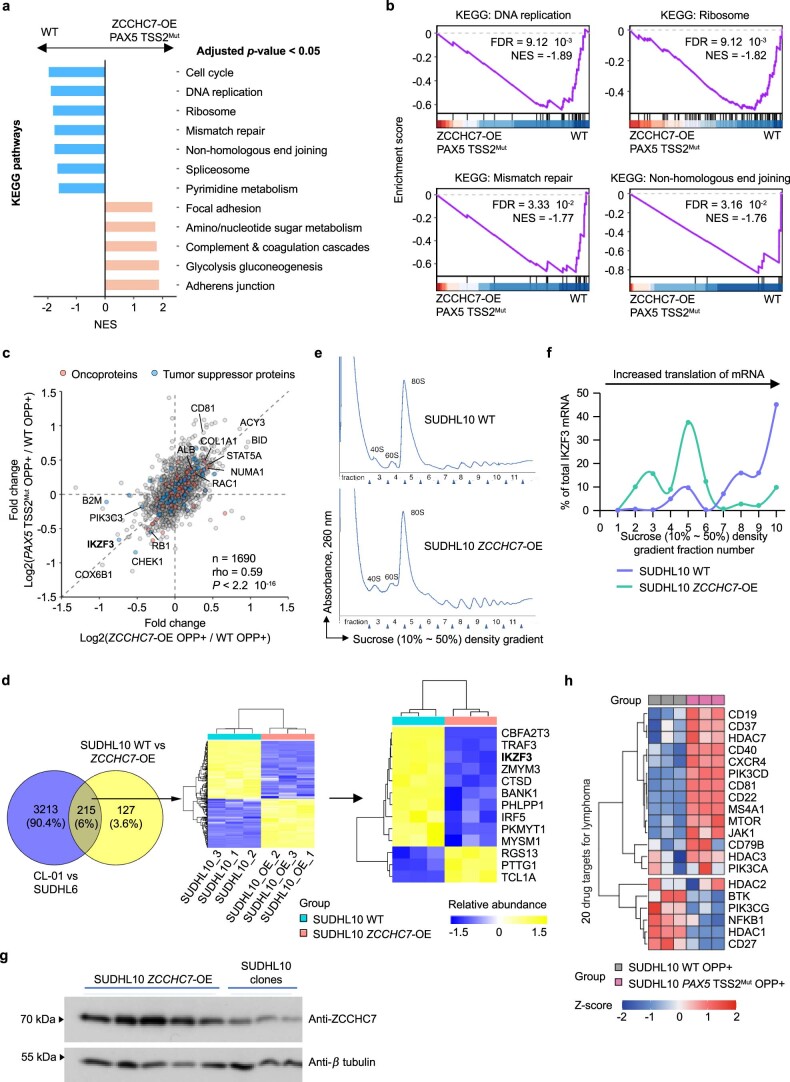
Proteomic changes resulting from ZCCHC7 alteration. **(a** and **b)** Ranked gene set enrichment analysis (GSEA) of O-propargyl puromycin-mediated identification (OPP-ID) data using KEGG pathways collection. Genes (n = 5,176) corresponding to altered proteins were ranked in ascending order by two-way ANOVA test *P*-value of the genetic alteration factor on the x-axis in Fig. 7e. Pathways that show FDR adjusted *P*-value < 0.05 are shown in (**a**). The sign of normalized enrichment score (NES) suggests downregulation (negative NES) or upregulation (positive NES) in the SUDHL10 *ZCCHC7*-OE cells and SUDHL10 *PAX5* TSS2^Mut^ cells compared to SUDHL10 WT cells. GSEA shows downregulation of proteins in ribosome, DNA replication, mismatch repair, and non-homologous end joining pathways (**b**). **(c)** Fold changes of nascent proteins in *ZCCHC7*-OE or *PAX5* TSS2^Mut^ SUDHL10 cells compared to the wild type SUDHL10 cells. Each dot represents a protein with significantly higher abundance in OPP+ than OPP- in a two-way ANOVA (Fig. 7e). 1,690 proteins in total were captured via OPP and evaluated for nascent protein level. *P*-value and rho of Spearman’s correlation test between the x-axis and y-axis show a significant positive correlation between fold change of proteins in *ZCCHC7*-OE and PAX5 TSS2^Mut^. Tumor suppressor proteins including IKZF3, CHEK1, etc show decreasing translation in both *ZCCHC7*-OE cells and *PAX5* TSS2^Mut^ cells. **(d)** Independent whole cell proteomics for triplicate cultures of CL-01 (non-neoplastic B cells), SUDHL6 (which overexpress *ZCCHC7* due to chromosomal translocation), SUDHL10 cells (no inherent alterations in *PAX5*-TSS2 or *ZCCHC7*) and *ZCCHC7*-OE (SUDHL10 cells overexpressing *ZCCHC7*). Of the 3428 proteins with different protein expression between SUDHL6 and CL-01, 215 showed alterations also observed in *ZCCHC7*-OE compared to SUDHL10. Some of these proteins are tumor suppressor proteins in B-NHL (for example IKZF3, TRAF3, etc.). **(e** and **f)** Polysome profiling of SUDHL10 WT and SUDHL10 ZCCHC7-OE (**e**) and analyses of IKZF3 mRNA distribution on the polysome (**f**). **(g)** *ZCCHC7* transgene was introduced into SUDHL10, and stable lines isolated. ZCCHC7 protein expression is compared in ZCCHC7-OE and the SUDHL10 parental cell line. Three biological replicates were performed. **(h)** Evaluation of nascent levels of 20 proteins targeted by available therapeutics. The expression heatmap of the proteins in SUDHL10 and SUDHL10 *PAX5*-TSS2^mut^ lymphoma cells is shown. Source data

Next, we wanted to investigate potential changes in nascent protein synthesis caused by the *PAX5*-TSS2 mutation. We performed O-propargyl puromycin-mediated identification (OPP-ID)^61^ of proteomic changes occurring within a short 2-h pulse of OPP. Briefly, in this assay, OPP permeates cells and labels nascent elongating polypeptides, which are then captured by click chemistry on streptavidin beads to allow for the identification of global changes in active/nascent protein translation (Fig. 7a). We performed three replicates of OPP-ID in SUDHL10 cells and those overexpressing *ZCCHC7* (Fig. 7b) or with *PAX5*-TSS2^m^^ut^ incorporation (Fig. 7c; robustness highlighted in PCA plot in Fig. 7d, with detailed OPP proteomics source data provided in Supplementary Table 5). We observe that de novo translation of many proteins is suppressed or stimulated as a consequence of the DHL-associated *PAX5* promoter mutation and ensuing ER as well as upon overexpression of *ZCCHC7*, and that many alterations in protein translation are common to these two conditions (Fig. 7e). Heatmaps demonstrating groups of proteins with altered synthesis and associated gene ontology enrichment analyses are provided in Extended Data Fig. 10a–c, suggesting effects on protein synthesis, DNA damage/mutational repair and processing, as well as other cellular mechanisms. Indeed, a group of oncogenes are translated rapidly (Fig. 7e) while the translation of several tumor suppressors is attenuated (Fig. 7e), including IKZF3 (which was also found to be decreased in steady-state protein level analyses (Extended Data Fig. 10d)). Using polysome analyses (Fig. 7f and Extended Data Fig. 10e), to fractionate mRNAs based on their translational efficiency^62^, we confirm that *IKZF3* mRNA indeed has lower translational efficiency in both *PAX5*-TSS2^mut^ lymphoma cells (Fig. 7g) and in *ZCCHC7*-OE lymphoma cells (Extended Data Fig. 10f,g), compared to parental SUDHL10 lymphoma cells. Thus, we postulate that increased ZCCHC7 expression in lymphoma cells results in altered kinetics of 5.8S rRNA biogenesis, driving changes in protein synthesis and consequently remodeling of the lymphoma proteome. Interestingly, some of the nascent polypeptide changes seen in *PAX5*-TSS2 mutant cells via the OPP-ID translation assay identify molecular targets of currently available therapeutics (Extended Data Fig. 10h)

## Discussion

The critical availability of multiple patient-derived paired longitudinal samples representing the transformation of FL to DHL provided a unique opportunity to answer an important question about the timing of SE mutations that occur in DLBCL/DHL. Furthermore, we demonstrated the consequences of these changes with respect to lymphoma biology. Our findings clarify the location of aSHM occurring in lymphoma transformation, with most of the observed mutations occurring near promoter-proximal H3K4me3-marked regions of lymphoma-related genes embedded in clusters of H3K4me1- and H3K27ac-marked SEs. The presence of mutations in promoter regions led us to perform studies that ultimately revealed a mechanism by which mutations in noncoding gene regulatory elements, such as SEs and associated promoters, can alter gene expression in lymphomas in unexpected ways, for example, via changes in the transcription of neighboring genes as opposed to simply altering transcription of cognate genes as expected. We demonstrate this phenomenon at the *PAX5*/*ZCCHC7* locus; however, it is possible that AID-mediated mutations of other gene regulatory elements will lead to similar outcomes.

In addition to highlighting other consequential AID-mediated mutations in gene regulatory elements, future studies might further clarify the mechanisms and parameters causing AID-mediated mutations specifically to these non-Ig regions of the lymphoma genome. Of note, aSHM mutational sites do not always directly overlap H3K27ac sites, thus making it unlikely that these marks or the associated enhancers are sufficient for recruitment of AID during progression to DHL. Majority of aSHM sites are situated ±2 kb from genic TSSs present inside H3K4me3 hubs, indicating divergently transcribed promoters (with genic TSS or alternative genic TSS) embedded in SEs, and thus promoter-associated transcriptional, chromatin and local DNA topological properties are important for AID recruitment. In addition, properties of TADs and SEs surrounding promoters are likely to be responsible for the recruitment of AID protein and/or AID cofactors at aSHM sites.

At the FL stage, SE mutations occur at low frequencies, whereas at the DHL stage, the frequencies are much higher. Phylogenetic analyses of aSHM in our FL/DHL paired samples indicate divergent evolution of FL and DHL from a common progenitor cell^24,63^. Given that the sequenced FL and DHL are clonally related, our findings support a role for SE-associated aSHM predominantly occurring during progression from a shared or common progenitor to DHL.

Although *PAX5* has a well-established integral role in B cell development and lymphomagenesis, the adjacent gene, *ZCCHC7*, has not been the subject of the same attention in B-NHL. In our longitudinal cohort, we find that ZCCHC7 overexpression can be altered by copy number gain and/or ER. We also sequenced DHL cases with high ZCCHC7 expression based on immunohistochemical (IHC) staining and identified one DHL (P11) harboring *MYC-LPP* and *BCL2-IGH* translocations as well as an *MYC*/*PVT1-ZCCHC7* translocation. This translocation has been reported previously in a subset of DHL^50^ and is found in the SUDHL6 cell line that overexpresses *ZCCHC7* mRNA. Taken together, our results provide evidence of the following three different mechanisms of *ZCCHC7* overexpression in DHL: copy number gain, ER and translocation, and a potential role for ZCCHC7 overexpression in lymphoma cell survival. It remains to be determined whether the identified mechanisms underlie transformation of FL in general and if they also foster the progression of other types of low-grade B cell lymphomas.

Finally, we reveal the cellular function of ZCCHC7 and the consequences of *ZCCHC7* alteration in lymphoma cells. ZCCHC7 overexpression via copy number gain or via ER interferes with normal 5.8S rRNA processing in DHL, with the resultant accumulation of 5.8S + 40 pre-rRNA leading to critical rewiring of the lymphoma proteome. We also provide a mechanistic role for ZCCHC7 in 5.8S rRNA processing, potentially acting as a part of the human TRAMP-like complex and thereby regulating the 3′–5′ exo-RNase Exosc10 in 5.8S rRNA processing. In addition to direct perturbation of homeostasis of tumor suppressors and/or oncogenes, alterations in rRNA biogenesis might cause translational stress that in turn might impact lymphomagenesis/lymphoma transformation (as has been previously implicated in the development of other malignancies^64,65^). We expect that in addition to the role of ZCCHC7, other mechanisms leading to rRNA processing defects in lymphoma likely will be identified in the future, presenting potential opportunities for the treatment of aggressive lymphomas and/or secondary prevention of lymphoma transformation.

**Fig. 8:**
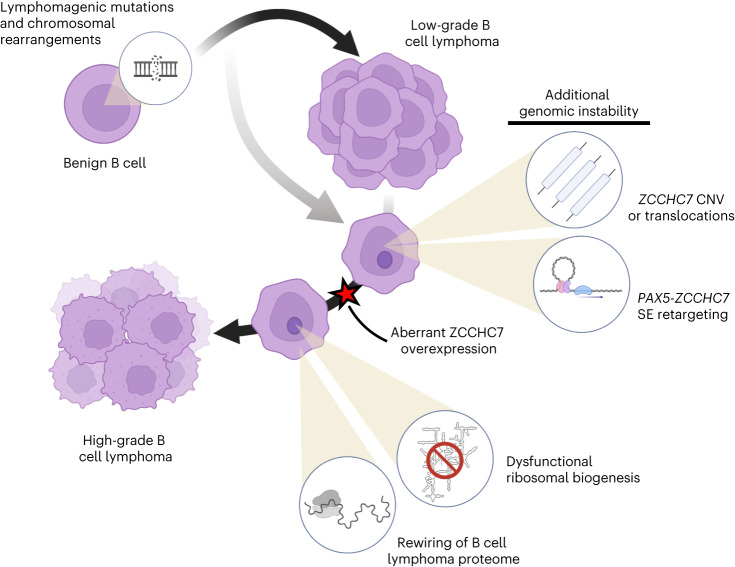
Schematic demonstrating the role of aSHM, *PAX5*/*ZCCHC7* alterations and altered protein synthesis in lymphoma transformation. A schematic representation of the contribution of aSHM to transformation from FL to DHL, along with various mechanisms of *PAX5*/*ZCCHC7* locus alterations that lead to aberrant rRNA processing and rewiring of lymphomagenic protein synthesis (created with BioRender.com).

In summary, our study highlights mechanisms by which gene and protein expression are broadly altered in lymphoma, via ER and effects on protein synthesis due to aberrant ribosome biogenesis, respectively (summarized schematically in Fig. 8). Future work will be required to understand the mechanisms through which AID is targeted to gene regulatory elements during lymphoma progression and to identify other cellular processes affected by ER in lymphoma cells. Finally, ZCCHC7-related changes to ribosome biogenesis present a mechanism through which the lymphoma proteome changes over time, with important implications for lymphoma biology and clinical management.

## Methods

### Case selection

The study was performed according to the principles of the Declaration of Helsinki and in compliance with protocols approved by the Institutional Review Boards (IRB) of Columbia University and the University of Pittsburgh. For samples that underwent WGS, departmental databases at CUIMC and UPMC were searched for longitudinal specimens representing DHL preceded by FL, diagnosed in the same individual and during the past 17 years. Samples from eight patients (four female and four male, ages 55–79 years, median 68 years), including formalin-fixed paraffin-embedded (FFPE) tissue (18 samples) or archival DNA extracted from fresh tissue for purposes of clinical molecular testing (five samples), were retrieved for WGS studies. When available, nontumor tissue or DNA from the same patients also was identified and retrieved from pathology archives (6/8 patients). Additionally, fresh tumor tissue from the two unpaired DHL found to demonstrate the highest degree of ZCCHC7 expression based on IHC staining of TMAs (described below) was retrieved for WGS. For samples used in TMA creation and immunohistochemistry, the pathology department database at CUIMC was searched for specimens representing FL, DLBCL and DHL, including paired specimens representing longitudinal FL/DLBCL samples from the same individual, and diagnosed over the past 15 years. All selected cases fulfilled the morphologic, immunophenotypic and cytogenetic features of FL, DLBCL and high-grade B cell lymphoma with MYC and BCL2 rearrangements according to the current WHO classification^66^. Clinical information and laboratory data for each individual were obtained through review of electronic health records. The study was carried out using de-identified, residual banked and archival tissue/nucleic acids originally collected for clinical diagnostic purposes and remaining after completion of diagnostic work-up. The requirement for informed consent was waived, as approved by the IRB, because the specimens used in this study are from patients who were diagnosed with an aggressive disease associated with a high mortality rate, many of whom were deceased at the time of the study, and whose residual diagnostic specimens were used retrospectively years after diagnosis. Obtaining informed consent would not be practical. Furthermore, no intervention was performed, and the study involved no more than minimal risk to the subjects.

### Creation of TMAs

Hematoxylin and eosin-stained sections derived from the paraffin blocks containing DLBCL and DHL were examined, and representative areas of interest were identified. Sector maps were designed using Microsoft Excel spreadsheets to identify the location of each specimen on the array blocks. Specifically, 33 DLBCL (15 female and 18 male, ages 12–85 years, median 71 years), 9 DHL (six female and three male, ages 42–76 years, median 66 years) and one each of low-grade FL (68 years, female), benign lymph node, tonsil and spleen were sampled in triplicate using 2 mm tissue cores. The DHL cases in the TMA and stained by IHC were distinct from those sequenced in the longitudinal cohort; however, a DHL in the TMA that showed high expression of ZCCHC7 based on IHC, from patient 11 (Extended Data Fig. 8a), was subsequently sequenced and analyzed separately from the main cohort. Each of the three lymphoma TMA blocks created included at least two samples (in triplicate) of benign lymphoid tissue controls. TMAs were created in the Experimental Molecular Pathology Core Facility of the Herbert Irving Comprehensive Cancer Center of Columbia University using a Beecher Instruments Manual Tissue Arrayer. Multiple four-micron sections from the tissue array blocks were cut and placed on charged polylysine-coated slides. These sections were used for IHC staining.

### IHC staining and scoring of slides

IHC staining of TMA and lymphoma tissue sections was performed using standard methods with a polyclonal antibody against ZCCHC7 (Novus Biologicals, NBP1-89175). Stained sections were evaluated by a hematopathologist (R.J.L.-N.) and assigned an IHC *H* score. *H* score = 3 × percentage of strongly positive cells + 2 × percentage of moderately positive cells + 1 × percentage of weakly positive cells.

### WGS

DNA was extracted from tissue samples using the QIAamp mini kit or the QIAamp FFPE kit (Qiagen) according to the manufacturer’s instructions. Samples underwent library preparation and sequencing on the Illumina Hi-Seq (8/10 patients, 19/26 samples) or DNBseq (2/10 patients, 7/26 samples) platform. Briefly, libraries sequenced on the Illumina platform were prepared using the NEBNext Ultra DNA Library Prep Kit according to the manufacturer’s recommendations. Libraries were sequenced on the Illumina HiSeq using 2× 150 bp paired-end configuration. Image analysis and base calling were carried out using HiSeq Control Software. Libraries sequenced on the DNBSeq by BGI technology were prepared using BGI’s in-house library preparation kit according to the manufacturer’s instructions. Libraries were sequenced on the BGISEQ-G400 platform.

### Antibodies

Antibodies (dilutions in parentheses) for western blot (WB) and indirect immunofluorescence (IF) were as follows: rabbit polyclonal anti-ZCCHC7 (Novus Biologicals, NBP1-89175; 1:500 for IF), rabbit polyclonal anti-ZCCHC7 (ABclonal, A28251; 1:1,000 for WB), mouse monoclonal anti-GAPDH (Proteintech, 60004-1-Ig; 1:20,000 for WB), Alexa Fluor 647 donkey anti-mouse IgG (Thermo Fisher Scientific, A-31571; secondary 1:500 for IF), Alexa Fluor 488 donkey anti-rabbit IgG (Thermo Fisher Scientific, A-21206; secondary 1:500 for IF), IRDye 800CW donkey anti-Mouse IgG (Licor, 926-32212; secondary 1:10,000 for WB) and IRDye 680RD donkey anti-rabbit IgG (Licor, 926-68073; secondary 1:10,000 for WB).

### Indirect IF

In total, 500,000 cells of each cell type (SUDHL6 obtained from American Type Culture Collection (ATCC), CRL-2959 and CL-01 obtained from Novus Biologicals, NBP1-49595) were incubated in PBS at 37 °C on poly-l-lysine-coated coverslips before fixation in 4% paraformaldehyde in PBS for 20 min, permeabilization with 1% Triton X-100 in PBS for 5 min and blocking with 1% powdered milk in PBS (IF-blocking buffer) for 60 min. The cells were then incubated for 2 h with primary antibodies in IF-blocking buffer, washed and incubated for 1 h with secondary antibodies in IF-blocking buffer in the dark. This was followed by washing and nuclear staining with 4′,6-diamidino-2-phenylidone (1 µg ml^−1^ in PBS). Coverslips were mounted on glass slides using ProLong Diamond Antifade Mount (Thermo Fisher Scientific, P36962). Spinning-disk confocal microscopy was performed on a Nikon TiE Eclipse inverted microscope (Nikon) equipped with a CSU-X1 spinning-disk unit (Yokogawa) and controlled with NIS Elements software (Nikon). A ×100/1.45 Plan Apo Lambda objective lens was used (Nikon). Fluorescence was excited with lasers at 405, 488, 561 and 647 nm, and emission was collected through standard filters for blue, green, red and far-red fluorophores. Z-stack images in 200-nm steps were acquired with a Zyla 4.2 CMOS camera (Andor Technology). Maximum projections were generated using ImageJ (National Institutes of Health (NIH)). Images for figures were cropped and adjusted using Photoshop (Adobe). To compare the different cell images, all images within the same panels and of the same antigens were acquired and adjusted identically.

### Generation of Exosc10-overexpressing SUDHL10 cells

SUDHL10 cells (CRL-2963) were purchased from the ATCC, cultured in the laboratory and modified through HDR to form the SUDHL10 *PAX5*-TSS^m^^ut^ cell line, as described elsewhere in this paper. The HDR cells were transduced with a plasmid purchased from GeneCopoeia expressing human Exosc10 (transcript variant 1)-IRES2-mCherry-IRES-puromycin (EX-G0202-Lv213) or the empty vector without the Exosc10 transcript (EX-NEG-Lv213) using a Lonza 4D nucleofector. Forty-eight hours post-transfection, the cells were analyzed on a Becton Dickinson (BD Biosciences) SORP FACSAria running BD FACSDiva software in the Columbia University Stem Cell Initiative Flow Cytometry Core Facility. Forward scatter (FSC-A) and side scatter (SSC-A) were used to gate on live cells, followed by gating on singlets through forward scatter area relative to forward scatter height (FSC-A versus FSC-H). A distinct population of mCherry-expressing cells was observed, described as ‘Exosc10-OE’ in Figs. 6f and 7b–e and Extended Data Figs. 9d and 10a–g for those receiving the Exosc10-expressing plasmid. These cells then were subjected to western blot.

### Pre-rRNA processing analysis in lymphoma cells

Five microliters of RNA were separated by migration on high-resolution denaturing acrylamide (for low-molecular-weight RNA analysis) or agarose (for high-molecular-weight RNA analysis) gels, and the gels were transferred to a nylon membrane and probed with radioactively labeled oligonucleotide probes (see ref. ^60^ for details). Mature rRNAs were visualized by ethidium bromide staining and northern blot probing. The probe sequences are shown in Supplementary Table 10.

Transcripts of interest were depleted for 3 d in HeLa cells (obtained from ATCC, CCL-2) transfected by RNAiMax (Invitrogen, 13778030) with silencers (used at 10 nM final concentration). ZCCHC7 was overexpressed following transfection with construct CMVp-hZCCHC7-Flag-IRES-GFP. The residual levels of ZCCHC7 after depletion (none was detected) or overexpression were established by western blotting with a specific antibody (ABClonal, A18251). As loading control, blots were probed for β-actin (Santa Cruz Biotechnology, SC-69879).

### 4C-seq protocol

To investigate chromatin interaction patterns of the *PAX5* promoter region, we analyzed the Hi-C heatmap from published Hi-C (GSE63525_GM12878_insitu_DpnII^67^) and HiChIP (GSE80820 (ref. ^68^)) data from the human B lymphocyte cell line, GM12878. We focused on two interacting regions around the *ZCCHC7* promoter locus that were found to strongly interact with the *PAX5* promoter. We selected one of the two strongly interacting regions for use as a bait after evaluating the efficiency of the primer sets available for 4C studies.

4C-seq^47,69^ was carried out as follows: after crosslinking of 10^7^ cells with 2% formaldehyde, HindIII (NEB, R3104S) was added to extracted nuclei, followed by overnight incubation, heat inactivation and washing with 1× T4 DNA ligase buffer (NEB, M0202S). The samples were resuspended in a 1.2 ml ligation mix (1× T4 DNA ligase, 1× BSA, 50 µl 20% Triton X-100, 5 µl T4 DNA ligase, H_2_O to 1.2 ml) and allowed to incubate at room temperature while rotating for 4–5 h, with addition of more ligase after 2 h. Reverse crosslinking was performed by adding 15 µl Proteinase K (Viagen Biotech, 501PK; 20 mg ml^−1^), followed by an overnight 65 °C incubation in phenol:chloroform:isoamyl alcohol (Sigma-Aldrich, P2069-100ML). The precipitated DNA was resuspended in 450 µl H_2_O. Samples were then digested with 50U DpnII (NEB, R0543S), followed by heat inactivation and purification with phenol:chloroform:isoamyl alcohol and resuspension in ~120 µl H_2_O. 4C PCR was carried out using Phusion High-Fidelity DNA Polymerase (NEB, M0530S) and two sets of viewpoint primers containing the Illumina sequencing adaptor sequences (see sequences in Supplementary Table 10). PCR parameters were as follows: 98 °C for 30 s, 16 cycles (98 °C for 10 s, 60 °C for 30 s, 72 °C for 2 min), 72 °C for 10 min. The products were cleaned with 0.8× Ampure XP beads (Beckman Coulter, A63880) as indicated in the referenced protocol. Addition of indexes and enrichment of adaptors containing first PCR product were carried out according to the manufacturer’s instructions (NEB, E7600/E7645). After quality control using the Bioanalyzer DNA HS chip, libraries were pooled and loaded on the Illumina MiniSeq using the MiniSeq High Output Reagent Kit (Illumina, FC-420-1002; 150-cycles). Both SUDHL10 *PAX5*-TSS2 deletion and point mutation clones were compared to control SUDHL10 using the same 4C-seq protocol.

### Preprocessing of WGS data

Fastp (v0.23) was used in preprocessing FASTQ files. Low-quality reads containing over 40% poor-quality bases (base quality < 15) were filtered out. After removing adapters, clean reads with at least 45 bp were used for subsequent analyses. These clean reads were then mapped to hg38 and sorted by coordinates using BWA (v0.7.15) and SAMtools (v1.2), respectively. Picard (v2.23.9) was used to mark duplicates and generate bam files for mutation identification.

### Somatic mutation calling in longitudinal samples

Somatic mutations in tumor samples were identified with the mutation caller SAVI (v2.0)^70^. For patient samples with available nontumor DNA, all primary and recurrent tumor samples were simultaneously compared to matched nontumor DNA for the identification of candidate somatic mutations. Variants exclusively identified in tumor samples (that is, those with *P* *<* 10^−6^ by the empirical Bayesian method and with <2 supporting reads in nontumor samples) were then annotated using multiple databases including dbSNP (https://www.ncbi.nlm.nih.gov/snp/), gnomAD (https://gnomad.broadinstitute.org/) and TOPMed (https://topmed.nhlbi.nih.gov/). After eliminating known common SNPs, the remaining candidates were subsequently filtered by eliminating (1) variants in low-complexity regions; (2) variants supported by <5 high-quality reads or supported by reads with strong strand biases; (3) variants with low allele frequencies (<10% for P2-FL and P8A-FL due to low tumor purity, <20% for others); (4) variants with total read depth of <15 in nontumor DNA; (5) variants only supported by the edge of reads (the distance from variants to all supporting reads’ 5′/3′ <25% of the read length). For patients without associated nontumor samples (P4 and P5) only mutations that differed between FL and DHL were used in analyses related to somatic mutations. For the same samples, known hotspot mutations found in the COSMIC (https://cancer.sanger.ac.uk/cosmic) database were listed as potentially pathogenic alterations.

### Copy number variant (CNV) detection

CNVkit (v0.9.9)^71^ was used to detect CNVs. Only exonic regions were considered in the analysis. For patients with matched nontumor DNA (P1, P2, P6, P7, P8 and P9), tumor samples were compared to their matched reference. For others (P4 and P5), a generic copy number reference with neutral expected coverage was constructed in CNVkit and used for comparison. GISTIC (v2.0) was used to compute significant focal copy number variations.

### Structural variant (SV) identification

Manta (v1.4.0)^72^ was used to identify somatic rearrangements involving *BCL2* and *MYC*. The somatic SVs called from Manta were initially filtered with default settings and then manually screened using the Integrative Genomics Viewer (v2.7.2). The allele frequency was estimated based on supporting reads containing the breakpoints and whole read coverage of two loci.

### Epigenetic annotation

The CTCF, H3K4me3 and H3K27ac ChIP–seq data from SUDHL6 and OCI-LY-1 cell lines were downloaded from ENCODE (ENCSR125DKL, ENCSR494LJG, ENCSR307DQT, ENCSR072EUE, ENCSR422JNY and ENCSR597UDW). The H3K4me1 ChIP–seq data are only available for OCI-LY-1 (ENCSR184QUS). SE regions were determined from the H3K27ac data of SUDHL6 and OCI-LY-1 via the ROSE method^67^. The TAD bed file, TAD boundary bed file, compartment information and Hi-C matrix data of the GM12878 cell line were downloaded from ref. ^73^ and used in the mutational annotation.

### Identification of potential ER gene pairs in the B cell lymphoma genome

A TAD annotation bed file generated from Hi-C data of the GM12878 cell line was used to narrow down potential ER gene pairs. Any two genes with both of their promoters located within the same TAD were considered as a gene pair with ER potential. Using published transcriptome data of 11 DLBCL cell lines^34^, expression profiles of the 19,676 gene pairs were investigated. These gene pair candidates were then ranked by the *P* values that represent ranking consistently high (or low) on multiple lists including the rank of mean expression of gene A, the rank of mean expression of gene B and the rank of Spearman’s correlation coefficient between gene A and gene B. These asymptotic *P* values were estimated via the central limit theorem using the ‘rankPvalue’ function of the WGCNA (v1.72-1) R package. In addition, all gene pairs were annotated with data regarding hypermutation in our DHL cohort and from ref. ^34^ (*n* = 39 DLBCL), as well as with data regarding gene pairs previously reported in ref. ^44^ to experience ER. Finally, by filtering out nonhypermutated gene pairs, the prioritized potential ER gene pairs affected by aSHM in the B cell lymphoma genome were acquired.

### Statistics and reproducibility

All statistical tests were computed with R v4.2.0, and details of statistical tests are indicated in all figures and corresponding figure legends. This is a retrospective study of all available FL/DHL pairs that were collected as part of diagnostic work in the University of Pittsburgh Medical Center and Columbia University Medical Center. Thus, no statistical test was used to predetermine sample size. One patient named ‘P3’ developed FL and DHL at the same time point, which may represent an early transformation. P3 was excluded from all analyses to remove any confusion.

Patients ‘P4’ and ‘P5’ were excluded from all somatic mutation burden analyses due to lack of matched nontumor DNA data. The experiments were not randomized. The investigators were not blinded to allocation during experiments and outcome assessment.

### Additional software used

STAR (v2.7.3a) was used to map RNA-seq data. FeatureCounts (v2.0.0) was used to calculate gene expression levels. Bedtools (v2.26.0) was used to do genome annotation. ProteinPaint was used to draw lollipop mutation diagrams. WashU Epigenome Browser (v54.0.4) was used to visualize Hi-C matrix data. PROMO (v3.0.2) was used for transcription factor binding motif analysis. CTCFBSDB (v2.0) was used to predict potential CTCF binding sites on a given sequence. Arriba 2.3.0 was used to calculate gene fusion events via RNA-seq data. R package clusterProfiler (v4.4.4) was used to do gene set enrichment analysis.

### Reporting summary

Further information on research design is available in the Nature Portfolio Reporting Summary linked to this article.

## Online content

Any methods, additional references, Nature Portfolio reporting summaries, source data, extended data, supplementary information, acknowledgements, peer review information; details of author contributions and competing interests; and statements of data and code availability are available at 10.1038/s41588-023-01561-1.

### Supplementary information

Supplementary Information Supplementary Methods and Figs. 1–4.

Reporting Summary

Supplementary Tables 1–10 Supplementary Tables 1–3: Detailed lists of all genomic alterations, including point mutations, SV and CNV seen in follicular lymphoma and DHL from the eight patients analyzed in this study (relevant to Figs. 1–5). Supplementary Table 4: DLBCL cell lines that harbor or lack genomic alterations of the PAX5/ZCCHC7 locus. Supplementary Table 5: Proteomics intensity data (in OPP-ID) of SUDHL10 relevant for Fig. 7 and Extended Data Fig. 10. Supplementary Tables 6 and 7: Detailed information about BCL2 rearrangements in follicular lymphoma and DHL from the eight patients in the cohort. Supplementary Tables 8 and 9: Detailed information about BCL2 rearrangements in follicular lymphoma and DHL from the eight patients in the cohort. Supplementary Table 10: Sequences of probes and oligonucleotides used.

### Source data

Source Data Figs. 1–5 and 7 and Extended Data Figs. 2, 3, 5, 8, 7 and 10 Source data for Figs. 1d–f, 2d, 3a,b, 4b,d, 5a,e and 7b,c,e,g and Extended Data Figs. 2d, 3b, 5c, 8a, 7e and 10a,c,f.

**Source Data Fig. 6** Northern blots for Fig. 6e,f. **Source Data Extended Data Fig. 9** Northern and western blots for Extended Data Fig. 9d. **Source Data Extended Data Fig. 10** Western blots for Extended Data Fig. 10g.

## Data Availability

Raw longitudinal WGS data from nine samples derived from five patients and collected before 2015 are available in dbGAP (phs003398.v1.p1). This IRB-approved study included a waiver of the requirement for informed consent. Per NIH policy, samples obtained after January 2015 cannot be uploaded to dbGAP without specific patient consent to do so. To access data from samples obtained after January 2015, investigators may contact the corresponding authors (U.B. or J.W.) and/or submit a request to the Columbia University Sponsored Projects Administration, via this form (https://cumc.co1.qualtrics.com/jfe/form/SV_29rqFAm9Dh4xX6Z), to obtain a data use agreement between Columbia University and the requesting institution. Following IRB approval of data sharing with each requesting institution, the data will be transferred electronically. Raw WGS data and RNA-seq data from 39 primary DLBCL cases and 11 DLBCL cell lines were downloaded from dbGaP—phs000235.v20.p6, and sequence read archive (SRA)—PRJNA523380. HiC data of GM12878 was downloaded from 4DN data portal (https://data.4dnucleome.org/) under accession 4DNES3JX38V5. 4C-Seq data was deposited in the Gene Expression Omnibus database with accession GSE210888. Raw WGS data and RNA-seq data from 29 DLBCL cell lines were downloaded from dbGaP—phs000328.v3.p1, SRA—PRJNA854968 and PRJNA523380. Proteomics data of SUDHL10 have been provided as source data in this paper. Source data are provided with this paper.

## Code availability

All code developed for this study is available at https://github.com/ForceField17/WatchDHL.
