# Supplementary material for: Noncoding mutations cause super-enhancer retargeting resulting in protein synthesis dysregulation during B cell lymphoma progression
Source: Nat Genet. 2023 Dec 4;55(12):2160–74. doi: 10.1038/s41588-023-01561-1 (PMC10703697; doi:10.1038/s41588-023-01561-1)
Supplement: Supplementary file 1 — Supplementary Methods and Figs. 1–4. [file 41588_2023_1561_MOESM1_ESM.pdf]

nature genetics

Article

https://doi.org/10.1038/s41588-023-01561-1

Noncoding mutations cause super-enhancer retargeting resulting in protein synthesis dysregulation during B cell lymphoma progression

In the format provided by the authors and unedited

SUPPLEMENTAL METHODS

Mass spectrometry data acquisition and analysis: Protein samples from human lymphoma cell lines were subjected to reduction and alkylation using 5mM DTT and 14mM iodoacetamide, respectively, followed by overnight digestion with trypsin. Peptide samples were desalted and subjected to LC-MS/MS using a Thermo Fisher Scientific EASY-nLC 1200 coupled on-line to a Fusion Lumos mass spectrometer (Thermo Fisher Scientific). Peptide separation was carried out on an in house packed 100 µm x 15 cm chromatography column (ReproSil-Pur C18-AQ, 3 µm, Dr. Maisch GmbH, Germany) using buffer A (0.1% formic acid in LC-MS grade water) and buffer B (0.1% formic acid in 80% acetonitrile) as mobile phases. For the first 30 minutes, peptides were separated using a gradient of 5-40% of buffer B followed by 40-80% buffer B over a period of 10 minutes. The flow rate was set to 400nL/min. Data independent acquisition (DIA) was performed using a Fusion Lumos mass spectrometer, where total MS scans were acquired in the Orbitrap mass analyzer over a range of 350-1400 m/z with a resolution of 120,000 at m/z 200. Using precursor isolation windows of 14 Thomson, DIA scans were performed to cover a range of 350-975. Fragmentation of isolated precursors by quadrupole was done using high energy collision dissociation (HCD) with a normalized collision energy of 27. Acquisition of MS/MS scans was done by Orbitrap mass analyzer with a resolution of 15,000 at m/z 200. The automatic gain control (AGC) target value for both MS and MS/MS was set to 3e6, with maximum ion injection time of 50ms for MS and 22 ms for MS/MS scans.

Protein identification and label free quantification was performed using the DIA-NN computational proteomics platform (version 1.8.1) [1]. Mass spectrometry data were searched against the UniProt human protein database using default parameters for library prediction and search, where the identified proteins were filtered by applying 1% FDR. Differentially expressed proteins with a p-

value of < 0.05 and a fold change of 1.5 were considered to be significantly altered. Heat maps for the differentially expressed proteins were plotted with (https://www.bioinformatics.com.cn/en).

Nascent protein profile by O-propargyl puromycin-mediated identification (OPP-ID) of human lymphoma cell lines

Live cells were treated with 30 µM OPP or DMSO vehicle for 2 h at 37 °C. After treatment, cells were collected and suspended in lysis buffer containing 100 mM Hepes at pH 7.5, 150 mM NaCl, 1% NP-40, 2 mM PMSF, and complete EDTA-free protease inhibitor (Roche), and for the subsequent Cu(I)-catalyzed click reactions with 1% SDS, 100 µM biotin-azide, 1 mM TCEP, 100 µM TBTA, and 1 mM CuSO4. After 1.5 hours at room temperature, reactions were quenched with 5 volumes of cold acetone. Precipitated proteins were resuspended and desalted, followed by immobilization with magnetic streptavidin beads (Pierce) at 4°C overnight with slow rotation. Beads were cleaned and resuspended in 20 mM ammonium bicarbonate. Samples were subject to reduction and alkylation using 5 mM DTT and 20 mM iodoacetamide, followed by incubation with Lys-C (Wako) for 4 hours followed by trypsin (Promega) overnight at 37 °C. The peptides were desalted with C18 stage tip and resuspended in 0.1% formic acid for analysis by LC-MS/MS. LC-MS/MS analyses were performed on an UltiMate 3000 UPLC system coupled with an Exploris 480 Orbitrap mass spectrometer (Thermo Fisher Scientific). A RSLC C18 analytical column (75 µm × 250 mm, 1.6 µm, 120 Å) (Aurora, IonOpticks) was employed for LC separation. Mobile phases A and B consist of 0.1% FA in water and 0.1% FA in 80% ACN, respectively. A 150-min gradient at a flow rate of 300 nL/min and an initial 8% mobile phase B was used. Mobile phase B was increased to 32% at 120 min, 90% at 140 min and held for 10 min. Data were collected in data-dependent acquisition (DDA) mode. The top ten precursor ions with a charge state of 2+ or higher were fragmented by HCD. The MS1 Orbitrap resolution was set at 120,000, and the

maximum injection time was set at 50 ms. The MS2 Orbitrap resolution was set at 30,000, and the maximum injection time was set at 50 ms.

The acquired DDA data were searched against the omo sapiens UniProt database using SEQUEST (Proteome Discoverer 2.4, Thermo Fisher Scientific). Precursor ion mass tolerance and fragmentation tolerance were set at 10 ppm and 0.02 Da for the database search. The maximum number of modifications and missed cleavages allowed per peptide were four and three, respectively. Carboxyamidomethylation on cysteine was set as fixed modification. N-terminal acetylation and methionine oxidation were set as variable modifications. The identified proteins were filtered with a false discovery rate of 1%, and normalization was performed against the total peptide amount.

Mass spectrometry of ZCCHC7: Mouse ZCCHC7 fused FLAG tag (in pEZ-M46 vector from GeneCopia™) and control vector was over-expressed as a fusion protein in HEK293T cells (obtained from ATCC, CRL-1573) with the help of PEI transfection. After 48hrs of transfection, the supernatant was discarded and cells were washed with cold PBS 3 times, after which the cells were lysed (20mM Tris7.5, 150mM NaCl, 1mM EDTA, 1mM EGTA, 1% TritonX-100, 1mM PMSF, 1X protease inhibitor) for 2hrs at 4°C. The cell lysate was collected and centrifuged at 20,000g for 30 min at 4°C. The supernatant was collected and protein content estimated using the Bradford method. An equal amount of protein (1mg) was taken from the control and the ZCCHC7 samples and the volumes were adjusted accordingly with the lysis buffer. The lysates were precleared with equilibrated magnetic beads (Sigma 49664) for 1hr at 4°C on the IP rotator. The precleared lysate was then incubated with anti-FLAG® M2 magnetic beads (Sigma M8823) for O/N at 4°C on the IP rotator. The flow through lysate was separated using magnetic stands and the anti-FLAG® M2

magnetic beads were washed with cold PBS + (0.1%) Tween for a total of 6 washes using the magnetic stand. The anti-FLAG® M2 magnetic beads bound proteins were eluted using the elution buffer [50mM Tris7.5, 150mM NaCl, 0.1mg/ml FLAG (Sigma F4799) peptide, Protease inhibitor 1X]. The elution was aliquoted and stored at -80°C for Mass spectrometry analysis, and a small amount was used for demonstrating the presence of the ZCCHC7 protein using western blotting against ZCCHC7 (AB Clonal A18251). The samples for mass spectrometry were submitted to the Proteomics and Macromolecular Crystallography Shared Resource, Columbia University. The mass spectrometry data were analyzed using the Perseus software. The significantly enriched proteins with an FDR value of < 0.05 were selected for generating the ZCCHC7 interacting protein network using Cytoscape and STRINGS database and by annotating functionally enriched proteins.

Differential expression analysis of proteins

Protein intensity matrices of 18 samples from SUDHL10 WT, ZCCHC7-OE, and PAX5 TSS2^{Mut} were normalized by the variance stabilizing transformation method after removing 69 proteins that displayed a '0' value over five times. When comparing differences in protein abundance between two groups, F-test was conducted first for each protein to evaluate the equality of variance ($P > 0.05$). Then, a two-sided T-test was performed with equality of variance set by the F-test results in the previous step. Significantly upregulated or downregulated nascent proteins were selected by FDR adjusted $P < 0.05$ and empirical fold change.

Introduction of the *PAX5* promoter region mutation: By combining Homology Directed Repair (HDR) and the Cas9 system, we generated a cell line with nucleotides GC (9:37026315-37026315 (GRCh38)) mutated to AC. To initiate the HDR guided point mutation, the Cas9 targeted the cutting site as indicated by a sgRNA transcribed from the same plasmid that expresses the active

Cas9 enzyme. Then the HDR templates (82bp for both positive and negative strands) which are strictly complementary to the 40bp nucleotide sequences on both sides of the mutated nucleotides GC, except that the GC are replaced with AT, were used as templates for HDR. sgRNA/HDR-templates were obtained from IDT (sequences in Supplementary Table 5) SgRNA-FWD and sgRNA-REV were used to construct a sgRNA expression plasmid with pX458 (Addgene, plasmid #48138) as the backbone. sgRNA expression plasmid and HDR templates were cotransfected into SU-DHL10 cells using the Lonza kit (VPA-1010). GFP positive cells were sorted into 96-well plates. Clones were screened by digesting PCR product (using primers screen-FWD: 5'-TTG CCC TCG CCC TAA ATG-3' and screen-REV: CGA AGC TCC AGC AGT GTT T-3') from corresponding genomic DNA with MluCI (NEB, R0538). Those with digested bands (321bp and 400bp) were considered to be positive and confirmed by Sanger sequencing.

Deletion of the PAX5 promoter region

SUDHL10 WT cells (obtained from ATCC, CRL-2963) were co-electroporated with two pX458 vectors (Addgene plasmid 48138) one containing an Upstream annealed pair of gRNAs and a separate pX458 plasmid containing a Downstream annealed pair (sequences in Supplementary Table 5). 48h post-transfection, based on green fluorescence expression, the cells were single-cell sorted into 96 well tissue culture plates. Cells were allowed to divide until a sufficient cell density was reached that cells could be lysed and assayed by PCR to determine deletion of the appropriate locus (primer sequences in Supplementary Table 5). Clones indicating deletion of the locus then were selected for further investigation. To compare expression levels of proteins of interest, RNA was made using Tri Reagent (Sigma-Aldrich T9424) with the addition of glycogen. RNA was resuspended in DEPC-treated water and quantitated. 1 ug of RNA then was treated with DNaseI (Life Technologies 18068105) and subsequently re-quantitated. 500 ng of purified

RNA then were transcribed into cDNA using random hexamers and Superscript IV (Life Techologies 18091050) following the manufacturer's instructions. Quantitative cycling was performed on a Roche LightCycler 480 (primer sequences in Supplementary Table 5).

Generating a structure model of the human TRAMP-like complex

To generate a structure model of the human TRAMP complex, the predicted AlphaFold[2] (AlphaFold Monomer v2.0) structure of human ZCCHC7 (AF-Q8N3Z6-F1-model_v3.pdb) was trimmed of its low confidence residues using the program PyMOL[3] (Version 2.4.0). Post trimming, the remaining high confidence ZCCHC7 residues were aligned (command: align, PyMOL) with those of its homolog Air2p, using co-ordinates from the crystal structure of the polymerase core of the yeast TRAMP complex (3nyb.pdb)[4]. The aligned regions contain a conserved IWRxY sequence flanked by two zinc knuckle domains. The same strategy was used to align the predicted AlphaFold structure of human PAPD5 (AF-Q8NDF8-F1-model_v4.pdb) with its yeast homolog Trf4p (3nyb.pdb),[4] which ultimately yielded a structure model of the human TRAMP subcomplex (ZCCHC7/PAPD5). Next, sequence alignments were carried out using Clustal Omega [5] (Version 1.2.4), to identify conserved regions in ZCCHC7 and ZCCHC8, a factor present in the human NEXT complex[6,7]. The alignments identified two conserved sequence patches. One of these patches codes for a zinc knuckle in both protein structures (ZCCHC7-residues 345-365, ZCCHC8-residues 227-244) while the second corresponds to a helix in both the predicted (ZCCHC7-residues 384-395, AlphaFold prediction) and experimental structures (ZCCHC8-residues 109-129, cryo-EM structure of human NEXT, 7s7b.pdb[6]). These regions were used to align the predicted structure of the human ZCCHC7/PAPD5 dimer with ZCCHC8 within the human NEXT complex to generate a structure model of the human TRAMP-like complex (ZCCHC7/PAPD5/MTR4/RNA).

REFERENCES

1 Demichev, V., Messner, C. B., Vernardis, S. I., Lilley, K. S. & Ralser, M. DIA-NN: neural networks and interference correction enable deep proteome coverage in high throughput. *Nat Methods* **17**, 41-44, doi:10.1038/s41592-019-0638-x (2020).

2 Jumper, J. *et al.* Highly accurate protein structure prediction with AlphaFold. *Nature* **596**, 583-589, doi:10.1038/s41586-021-03819-2 (2021).

3 Delano, W. The PyMol Molecular Graphics System. *CCP4 Newsl. Protein Crystallogr.* (2002).

4 Hamill, S., Wolin, S. L. & Reinisch, K. M. Structure and function of the polymerase core of TRAMP, a RNA surveillance complex. *Proc Natl Acad Sci U S A* **107**, 15045-15050, doi:10.1073/pnas.1003505107 (2010).

5 Sievers, F. *et al.* Fast, scalable generation of high-quality protein multiple sequence alignments using Clustal Omega. *Mol Syst Biol* **7**, 539, doi:10.1038/msb.2011.75 (2011).

6 Puno, M. R. & Lima, C. D. Structural basis for RNA surveillance by the human nuclear exosome targeting (NEXT) complex. *Cell* **185**, 2132-2147 e2126, doi:10.1016/j.cell.2022.04.016 (2022).

7 Gerlach, P. *et al.* Structure and regulation of the nuclear exosome targeting complex guides RNA substrates to the exosome. *Mol Cell* **82**, 2505-2518 e2507, doi:10.1016/j.molcel.2022.04.011 (2022).

Patient	1	2	4	5	6	7	8	9
Age, sex	68F	63M	79F	74F	68M	68M	64F	55M
Grade of FL	1-2	1-2	1-2	3A	1-2	1-2	1-2	1-2
Site (other involved sites)	Inguinal LN	Abdominal LN (cervical, supraclavicular,axillary, mediastinal, retroperitoneal LNs)	Axillary LN (bone marrow)	Omentum (peritoneum, inguinal LN, pleura, bone)	Axillary LN (paratracheal and mesenteric LNs)	Mesenteric LN	Mesenteric LN, (supraclavicular, mediastinal, internal mammary, mesenteric, pelvic, and retroperitoneal LNs)	Axillary LN, (retroperitoneal and abdominal LNs)
FL therapy	R-Bendamustine	R-CHOP, R-Bendamustine, Ublituximab, Azacytidine-Romidepsin	Rituximab, R-CHOP, R-CVP	R-Bendamustine	Rituxumab	Rituxumab, ASCT	R-CHOP	R-Bendamustine
Time to transformation (months)	6	15	161	41	38	134	48	29
Site	Arm (bone and muscle)	Cervical LN	Axillary LN	Colon	Iliac mass	Abdominal LN	Paraspinal mass	Retroperitoneal LN
DHL therapy	R-CHOP	R-ICE	R-Bendamustine, radiotherapy	R-EPOCH	R-CHOP	R-EPOCH, CAR-T	R-ICE, CAR-T	R-CHOP, R-ICE, ASCT after CR
Alive/Dead/ Cause of death	Dead	Dead/ disseminated strongyloides	Dead/ Lymphoma	Alive	Dead	Alive	Alive	Alive

Abbreviations: LN lymph node; R- Rituximab; CHOP Cyclophosphamide, Doxorubicin, Vincristine and Prednisolone; CVP Cyclophosphamide, Vincristine and Prednisolone; ASCT Autologous stem cell transplant; EPOCH Etoposide, Prednisolone, Vincristine, Cyclophosphamide, and Doxorubicin; ICE Ifosfamide, Carboplatin and Etoposide; CAR-T Chimeric antigen receptor T-cell therapy

Supplementary Fig. 1. Clinical features of the cohort. Clinical information relating to the 8 patients whose FL and DHL genomes were analyzed in this study.

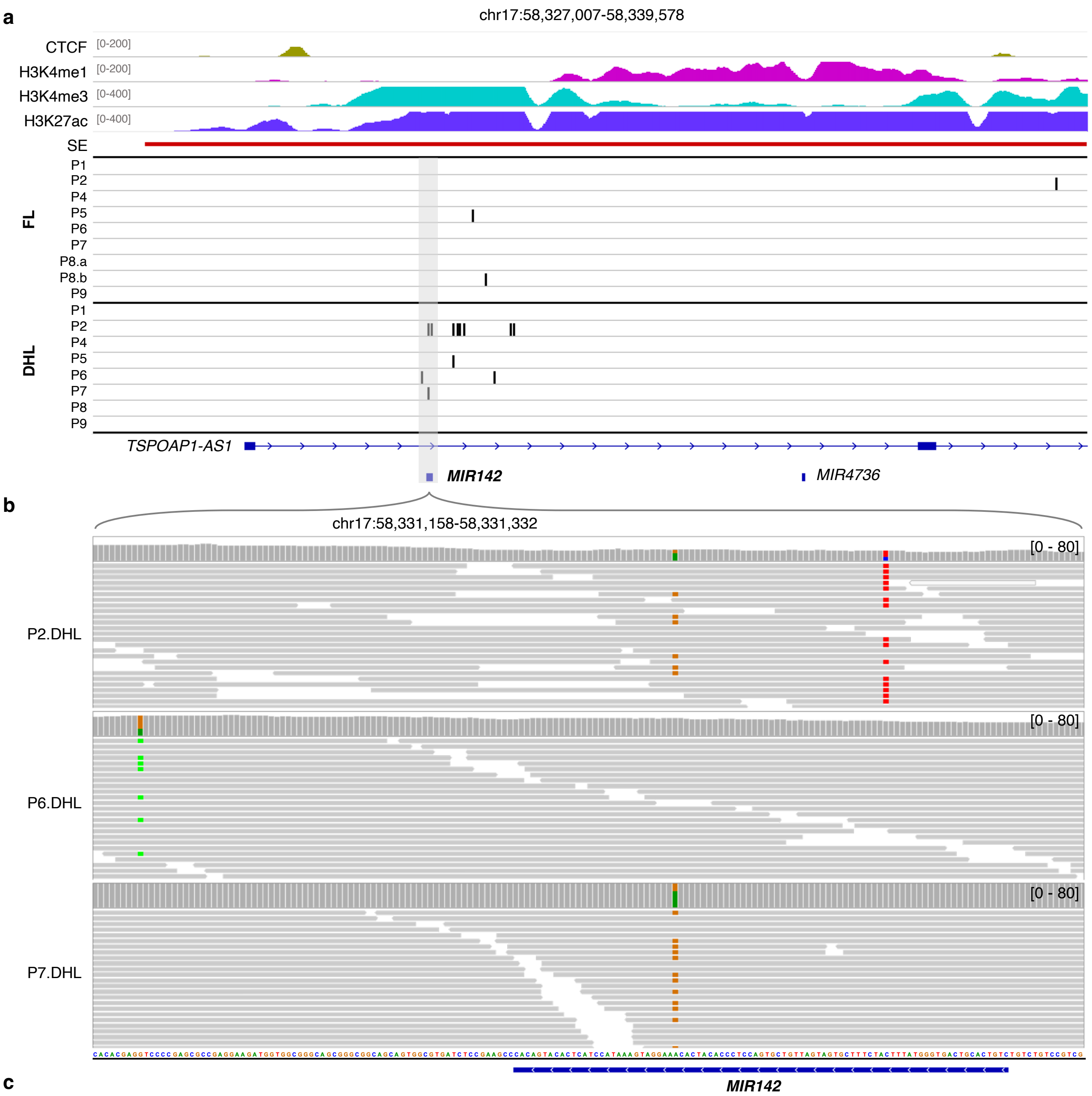

c

Case ID	Chr.	position	ref	alt	SNP ID	Gene	Normal A.F.	FL A.F.	DHL A.F.	WRCY signature
P6	chr17	58331166	G	A	-	-	0	0	0.34	False
P2	chr17	58331260	A	G	rs756348475	*MIR142*	0	0	0.33	False
P7	chr17	58331260	A	G	rs756348475	*MIR142*	0	0.14	0.54	False
P2	chr17	58331297	C	T	-	*MIR142*	0	0	0.58	True

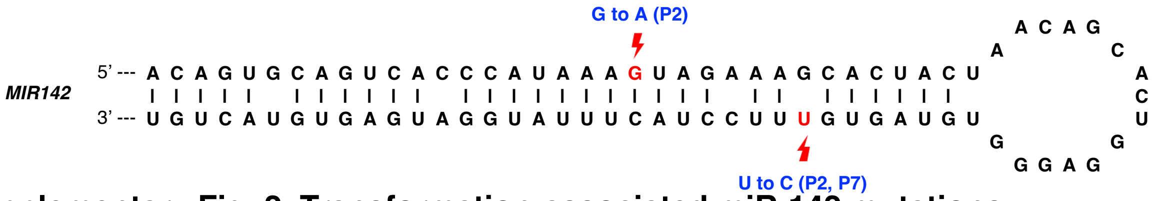

Supplementary Fig. 2. Transformation-associated miR-142 mutations
(a) Locations of recurrent transformation-associated mutations in miR-142 in relation to H3K27ac, H3K4me3, and other ChIPseq peaks. **(b)** Details of SNV acquired in miR-142 in patients 2, 6, and 7 upon transformation to DHL. **(c)** Table describing the features of the observed miR-142 mutations and schematic demonstrating the location of each mutation.

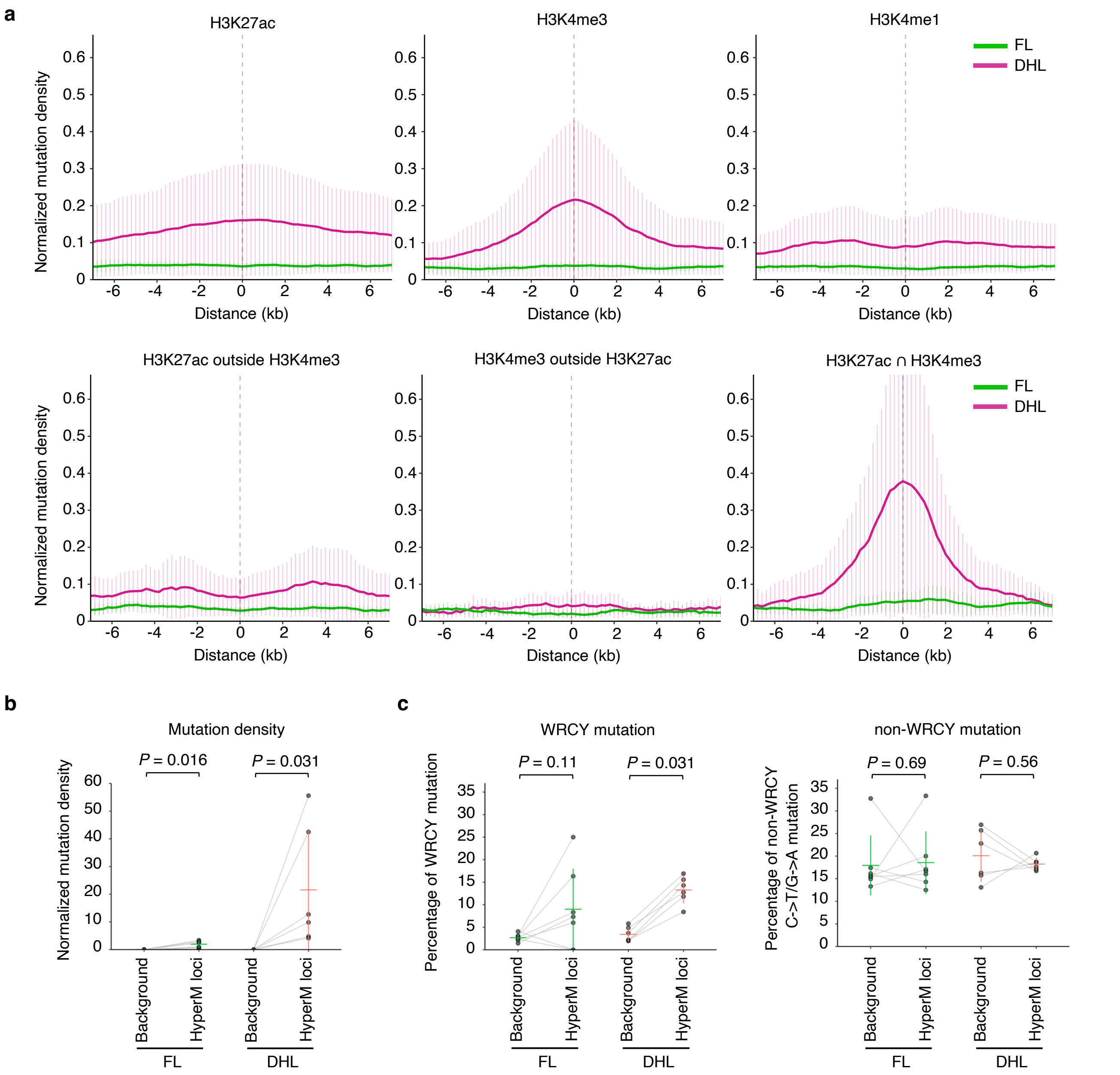

Supplementary Fig. 3. Narrow analyses of aSHM at superenhancers identifies promoters as targets. (a) aSHM focused on a narrower distribution of H3K4me3 and H3K27ac islands. SE-associated aSHM are mostly found within a +/- 2Kb window of $H3K4me3^{+}H3K27ac^{+}$ marks. **(b)** Comparison of normalized mutation density between hypermutated loci and the non-hypermutated background of the whole genomes for FL (n = 7 samples) and DHL (n = 6 samples). The hypermutated loci (regions containing >=4 mutations with an inter-mutational distance <= 1 Kb, and at least two tumors mutated) were identified using the rainfall plot in Figure 4a. The *P*-values were calculated using two-sided Wilcoxon signed-rank tests. The horizontal and vertical line in each group represent its mean value +/- its standard deviation. **(c)** Comparison of WRCY mutation frequency (left) and non-WRCY C->T/G->A mutation frequency (right) between hypermutated loci and non-hypermutated background of the whole genomes for FL (n = 7 samples) and DHL (n = 6 samples). Each pair of dots linked by a grey line represents a tumor sample. P-values were calculated using two-sided Wilcoxon signed-rank tests. The horizontal line in each group represents its mean value, and the corresponding vertical line represents its standard deviation bar. The results show a significant increase in AID-induced mutations with WRCY motif in the hypermutated loci of DHL, as compared to the background genome.

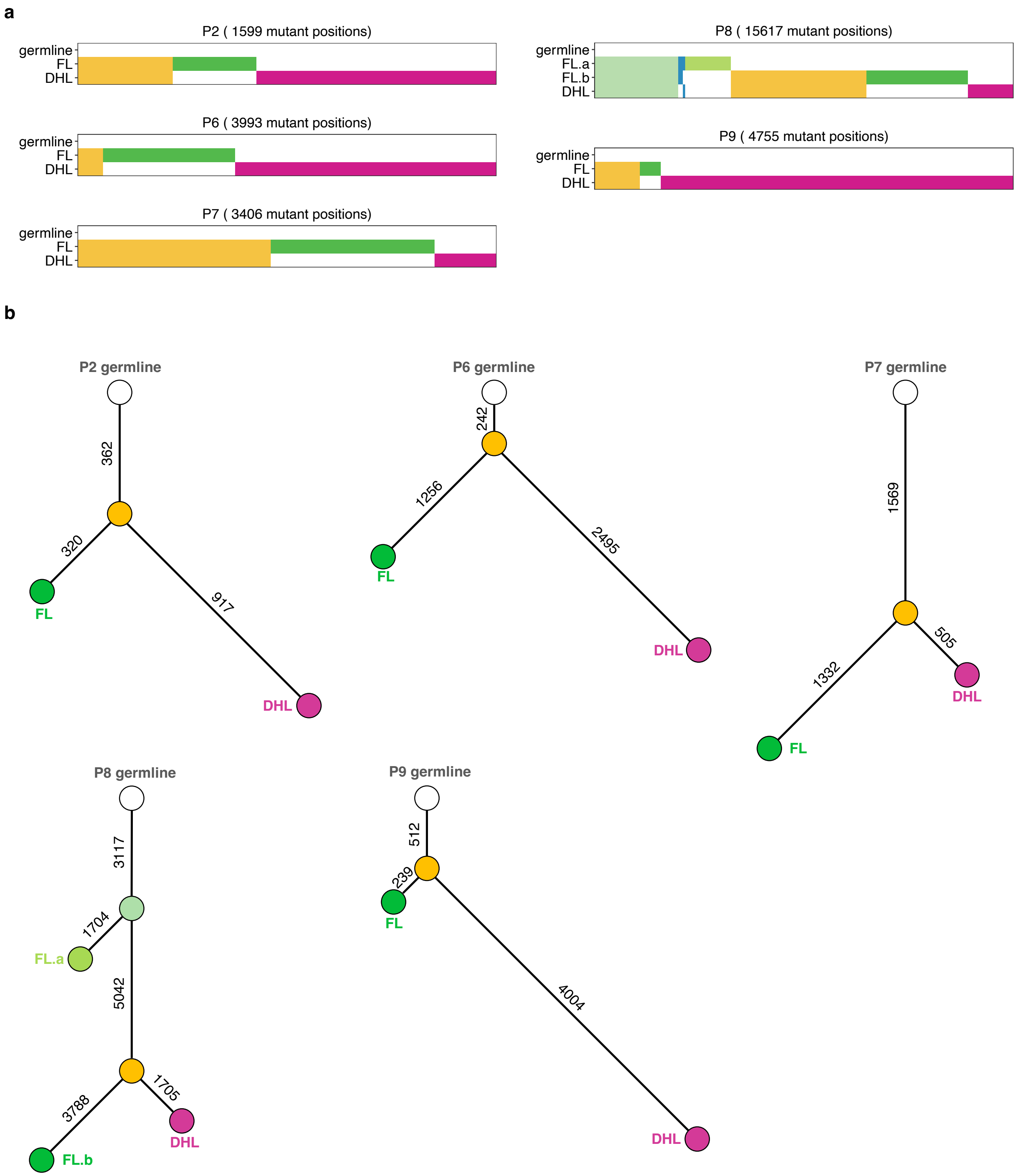

Supplementary Fig. 4. Tumor evolution analysis based on somatic point mutations.
(a) Heatmap plots describing the distribution of mutations with at least 15 sequencing reads of coverage from non-tumor and corresponding tumor samples, for each patient. P1, P4, and P5 are not included in this analysis due to lack or low coverage of non-tumor DNA sequences. **(b)** Phylogenetic trees of P2, P6, P7, P8, and P9 based on the mutational heatmap results summarized in (a).
