# Supplementary material for: Noncoding mutations cause super-enhancer retargeting resulting in protein synthesis dysregulation during B cell lymphoma progression
Source: Nat Genet. 2023 Dec 4;55(12):2160–74. doi: 10.1038/s41588-023-01561-1 (PMC10703697; doi:10.1038/s41588-023-01561-1)
Supplement: Supplementary file 2 — Reporting Summary [file 41588_2023_1561_MOESM2_ESM.pdf]

nature portfolio

Corresponding author(s): Uttiya Basu

Last updated by author(s): Aug 18, 2023

Reporting Summary

Nature Portfolio wishes to improve the reproducibility of the work that we publish. This form provides structure for consistency and transparency in reporting. For further information on Nature Portfolio policies, see our Editorial Policies and the Editorial Policy Checklist.

Statistics

For all statistical analyses, confirm that the following items are present in the figure legend, table legend, main text, or Methods section.

n/a	Confirmed	
☐	☒	The exact sample size (*n*) for each experimental group/condition, given as a discrete number and unit of measurement
☐	☒	A statement on whether measurements were taken from distinct samples or whether the same sample was measured repeatedly
☐	☒	The statistical test(s) used AND whether they are one- or two-sided<br>*Only common tests should be described solely by name; describe more complex techniques in the Methods section.*
☒	☐	A description of all covariates tested
☐	☒	A description of any assumptions or corrections, such as tests of normality and adjustment for multiple comparisons
☐	☒	A full description of the statistical parameters including central tendency (e.g. means) or other basic estimates (e.g. regression coefficient) AND variation (e.g. standard deviation) or associated estimates of uncertainty (e.g. confidence intervals)
☐	☒	For null hypothesis testing, the test statistic (e.g. *F*, *t*, *r*) with confidence intervals, effect sizes, degrees of freedom and *P* value noted<br>*Give P values as exact values whenever suitable.*
☒	☐	For Bayesian analysis, information on the choice of priors and Markov chain Monte Carlo settings
☒	☐	For hierarchical and complex designs, identification of the appropriate level for tests and full reporting of outcomes
☒	☐	Estimates of effect sizes (e.g. Cohen's *d*, Pearson's *r*), indicating how they were calculated

Our web collection on statistics for biologists contains articles on many of the points above.

Software and code

Policy information about availability of computer code

Data collection	HiSeq Control Software was used to collect fastq file in whole genome sequencing.
Data analysis	fastp v0.23;<br>BWA v0.7.15;<br>SAMtools v1.2;<br>Picard v2.23.9;<br>SAVI v2.0 (https://github.com/WangLabHKUST/SAVI);<br>CNVkit v0.9.9;<br>GISTIC v2.0;<br>Manta v1.4.0;<br>IGV v2.7.2;<br>WGCNA v1.72-1;<br>DIA-NN v1.8.1;<br>STAR v2.7.3a;<br>featureCounts v2.0.0;<br>bedtools v2.26.0;<br>R v4.2.0;<br>ProteinPaint (https://proteinpaint.stjude.org/);<br>WashU Epigenome Browser v54.0.4;<br>PROMO v3.0.2;<br>CTCFBSDB v2.0;<br>Arriba 2.3.0.<br>clusterProfiler v4.4.4;

AlphaFold Monomer v2.0;
Clustal Omega v1.2.4;
PyMOL v2.4.0;
SEQUEST (Proteome Discoverer 2.4, Thermo Fisher Scientific);
Cytoscape v3.10.0;
STRINGS v12.0.

For manuscripts utilizing custom algorithms or software that are central to the research but not yet described in published literature, software must be made available to editors and reviewers. We strongly encourage code deposition in a community repository (e.g. GitHub). See the Nature Portfolio guidelines for submitting code & software for further information.

Data

Policy information about availability of data

All manuscripts must include a data availability statement. This statement should provide the following information, where applicable:

- Accession codes, unique identifiers, or web links for publicly available datasets
- A description of any restrictions on data availability
- For clinical datasets or third party data, please ensure that the statement adheres to our policy

Raw longitudinal WGS data from 10 samples derived from 5 patients and collected prior to 2015 are available in dbGAP (phs003398.v1.p1). This IRB-approved study included a waiver of the requirement for informed consent, as obtaining informed consent would not be practical for the use of residual diagnostic tissue from this retrospectively-identified cohort of patients with rare and frequently fatal tumors. Per NIH policy, samples obtained after January 2015 cannot be uploaded to dbGAP without specific patient consent to do so. In order to access data from these samples, investigators may contact the corresponding author and obtain a data use agreement with Columbia University.
Raw WGS data and RNAseq data from primary DLBCL cases & 11 DLBCL cell lines were downloaded from dbGaP (phs000235.v20.p6).
HiC data of GM12878 was downloaded from 4DN data portal (https://data.4dnucleome.org/) under accession no. 4DNES3JX38V5.
4C-Seq data was deposited in GEO database with accession number GSE210888.
Raw WGS data and RNAseq data from 29 DLBCL cell lines were downloaded from dbGaP: phs000328, SRA: PRJNA854968, & SRA: PRJNA523380.
Outputs from the data analysis and source data are included in supplementary tables listed in the SI guide.

Field-specific reporting

Please select the one below that is the best fit for your research. If you are not sure, read the appropriate sections before making your selection.

☒ Life sciences ☐ Behavioural & social sciences ☐ Ecological, evolutionary & environmental sciences

For a reference copy of the document with all sections, see nature.com/documents/nr-reporting-summary-flat.pdf

Life sciences study design

All studies must disclose on these points even when the disclosure is negative.

Sample size	The total number of human subjects were limited by the number of FL/DHL paired samples we were able to acquire.
Data exclusions	This study was focus on FL to DHL transformation. To avoid any confusion, WGS data from one patient who developed FL and DHL at the same time point were excluded from all analyses.<br>Two patients were excluded from somatic mutation burden analyses due to lack of matched non-tumor DNA data.
Replication	4C-seq studies were performed 3 times. Tissue microarrays contained triplicate samples of each tumor/tissue sample. Mass spectrometry experiments were performed 3 times. Northern blot experiments to evaluate rRNA were performed 2 times. Flow cytometric experiments were replicated 3 times. Polysome profiling experiments were performed X times. OPP-ID studies were done with six biological replicates per group. Three replicates each were cultured with or without O-propargyl-puromycin (OPP+ and OPP-, respectively).
Randomization	Samples were not blinded and not randomized.
Blinding	experiments were not blinded except Fig. 4e

Reporting for specific materials, systems and methods

We require information from authors about some types of materials, experimental systems and methods used in many studies. Here, indicate whether each material, system or method listed is relevant to your study. If you are not sure if a list item applies to your research, read the appropriate section before selecting a response.

Materials & experimental systems

n/a	Involved in the study
☐	☒ Antibodies
☐	☒ Eukaryotic cell lines
☒	☐ Palaeontology and archaeology
☐	☒ Animals and other organisms
☐	☒ Human research participants
☒	☐ Clinical data
☒	☐ Dual use research of concern

Methods

n/a	Involved in the study
☒	☐ ChIP-seq
☒	☐ Flow cytometry
☒	☐ MRI-based neuroimaging

Antibodies

Antibodies used

Rabbit polyclonal anti-ZCCHC7 (1:500 for IF; 1:1000 for WB; Novus Biologicals NBP1-89175); rabbit polyclonal anti-ZCCHC7 (1:1000 for WB; ABclonal A28251); mouse monoclonal anti-GAPDH (1:20000 for WB; Proteintech 60004-1-Ig); Alexa Fluor 647 donkey anti-mouse IgG (secondary 1:500 for IF; Thermo Fisher Scientific A-31571); Alexa Fluor 488 donkey anti-Rabbit IgG (secondary 1:500 for IF; Thermo Fisher Scientific A-21206); IRDye 800CW donkey anti-Mouse IgG (secondary 1:10000 for WB; Licor 926-32212); IRDye 680RD donkey anti-Rabbit IgG (secondary 1:10000 for WB; Licor 926-68073).

Validation

Negative control cell lines were used to validate the ZCCHC7 antibody. Manufacturer used western blotting of the protein and showed it migrated at the right size. Validation by overexpression of ZCCHC7-Flag in HeLa and detection of the endogenous and the construct by WB (while only the endogenous ZCCHC7 is detected in un-transfected cells). The anti-GAPDH antibody was validated by the manufacturer via western blot of multiple human cell lines as shown on their website: https://www.ptglab.com/products/GAPDH-Antibody-60004-1-Ig.htm#publications

Eukaryotic cell lines

Policy information about cell lines

Cell line source(s)

CL01, SU-DHL6, SU-DHL10, CH12F3, 293T, HELA

Authentication

CL01, SU-DHL6, SU-DHL10, 293T, and HELA were purchased from ATCC. Whole genome sequencing of these cell lines have been performed to characterize them and the data is available publicly. CH12F3 is widely used in many labs that work in the B cell field. It can undergo class switch recombination from IgM to IgA (the only cell line that can do so)

Mycoplasma contamination

Cell lines were not tested for mycoplasma contamination

Commonly misidentified lines
(See ICLAC register)

No commonly misidentified lines from the ICLAC register were used.

Animals and other organisms

Policy information about studies involving animals; ARRIVE guidelines recommended for reporting animal research

Laboratory animals

For laboratory animals, report species, strain, sex and age OR state that the study did not involve laboratory animals.

Wild animals

N/A

Field-collected samples

N/A

Ethics oversight

Columbia University IACUC

Note that full information on the approval of the study protocol must also be provided in the manuscript.

Human research participants

Policy information about studies involving human research participants

Population characteristics

Biospecimens were derived from living and deceased adult (>18 years of age) patients representing both male and female sexes and mostly within the 5th-8th decades of life. Research subjects were selected for inclusion in the study based on their diagnosis of double hit lymphoma, follicular lymphoma, and/or diffuse large B cell lymphoma.

Recruitment

Participants were not recruited to the study. The study utilized remnants of diagnostic tissue and/or nucleic acids present within pathology archives. Research subjects were selected based on their lymphoma diagnoses.

Ethics oversight

The study was performed according to the principles of the Declaration of Helsinki and in compliance with protocols approved by the Institutional Review Boards of Columbia University and University of Pittsburgh.

Note that full information on the approval of the study protocol must also be provided in the manuscript.
