# Supplementary material for: Noncoding mutations cause super-enhancer retargeting resulting in protein synthesis dysregulation during B cell lymphoma progression
Source: Nat Genet. 2023 Dec 4;55(12):2160–74. doi: 10.1038/s41588-023-01561-1 (PMC10703697; doi:10.1038/s41588-023-01561-1)
Supplement: Supplementary file 5 — Source Data Fig. 6 Northern blots for Fig. 6e,f. Source Data Extended Data Fig. 9 Northern and western blots for Extended Data Fig. 9d. Source Data Extended Data Fig. 10 Western blots for Extended Data Fig. 10g. [file 41588_2023_1561_MOESM5_ESM.pdf]

Fig. 6e

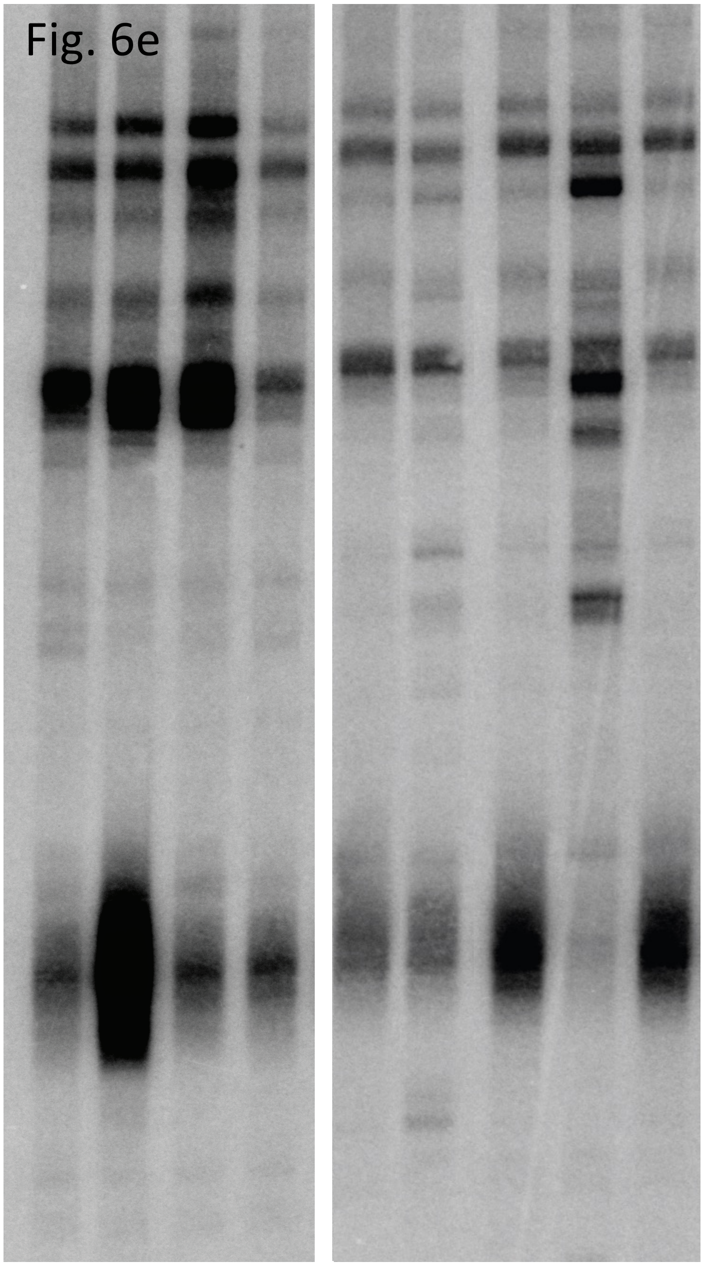

Fig. 6f

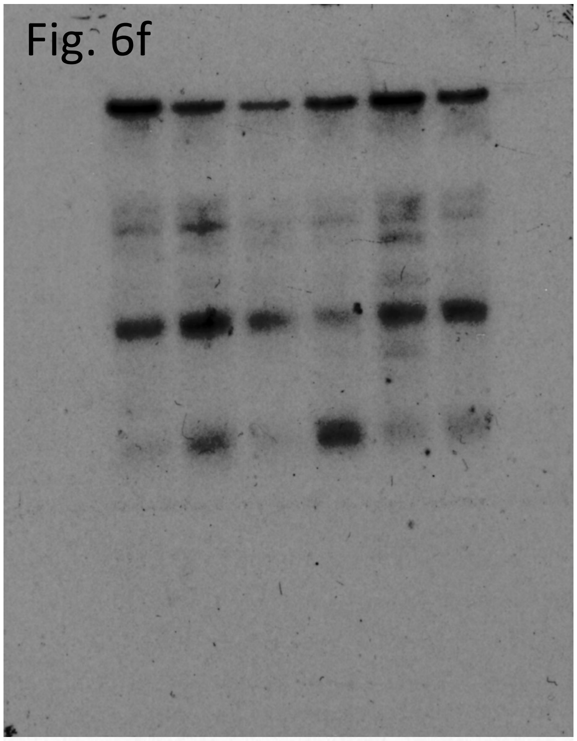

Fig. 6f

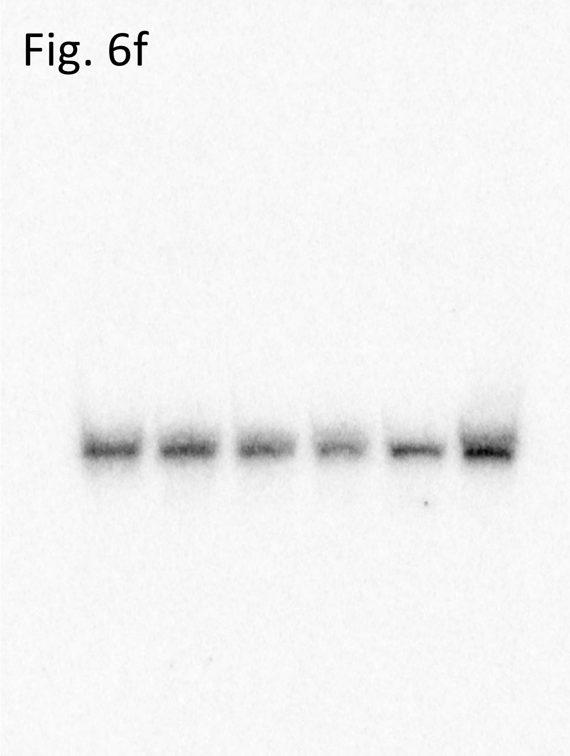

Source data for Fig. 6

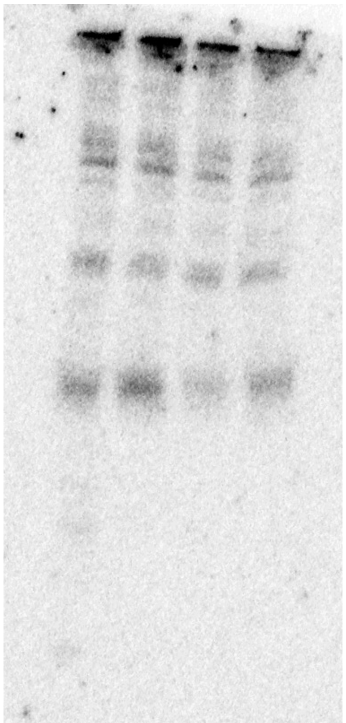

Extended Fig. 9d

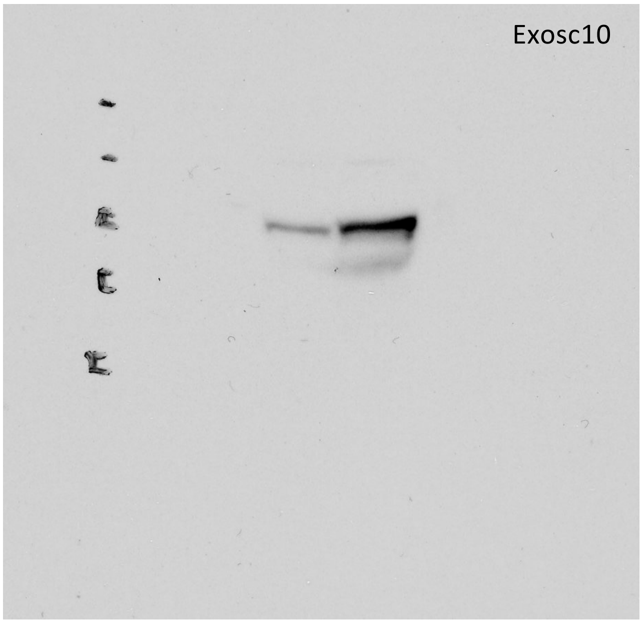

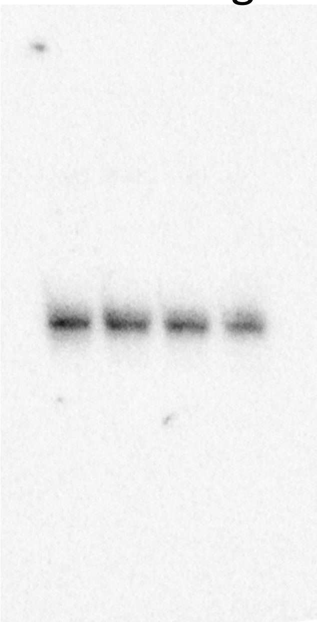

Extended Fig 9d

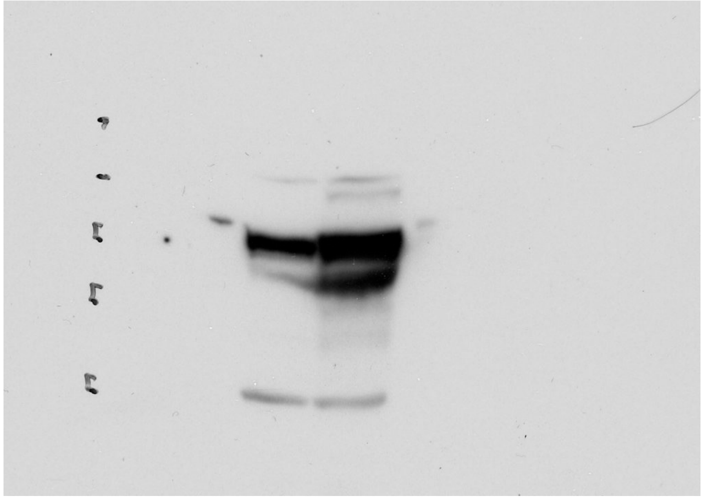

β tubulin (did not strip after Exosc10 acquisition since blot is clean in the B-tubulin size range)

Extended Fig 9d

Source data for Extended Fig. 9

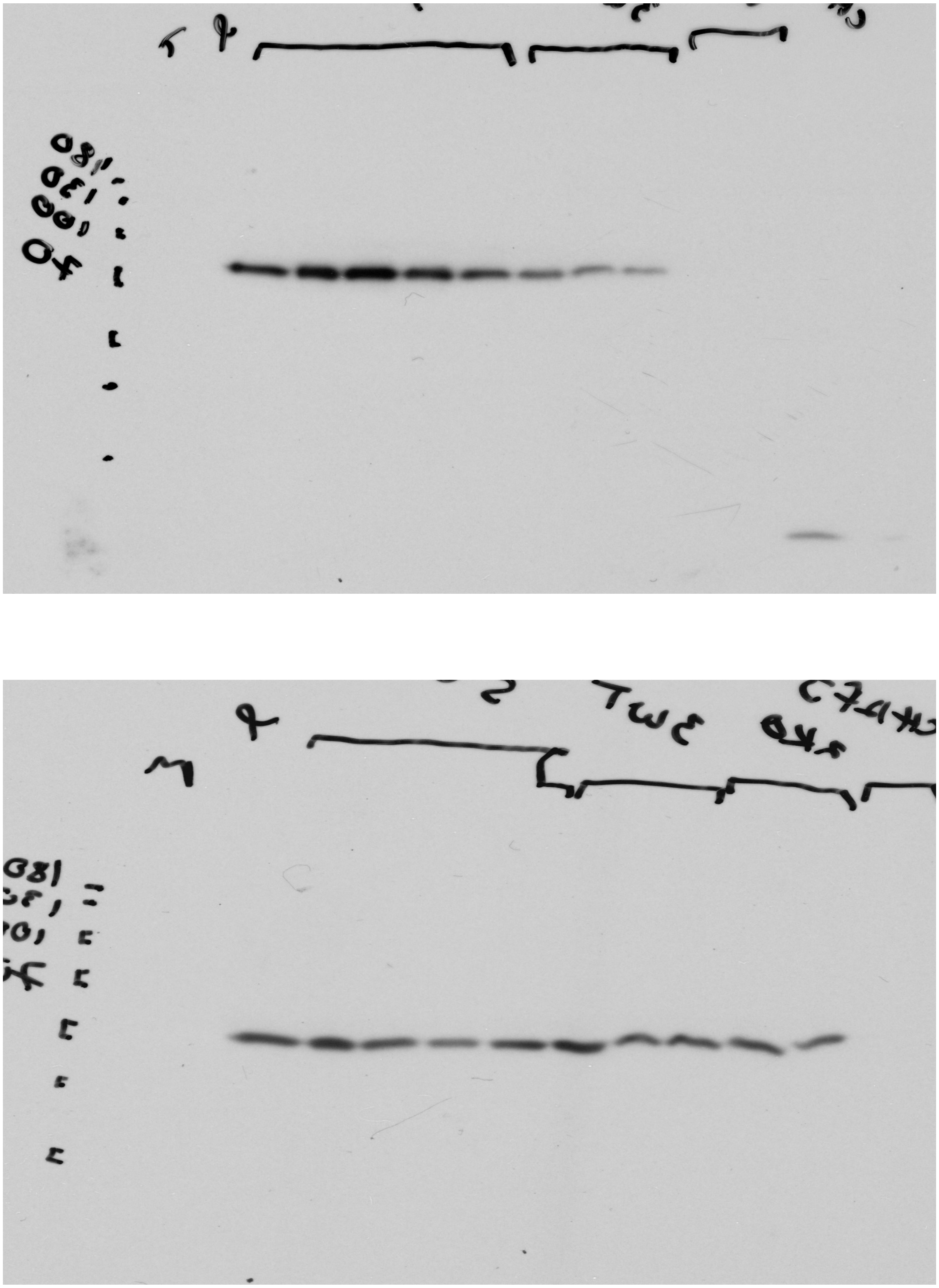

Extended Fig. 10g
